# Genomic analysis and functional properties of *Lactobacillus johnsonii* GJ231 isolated from healthy beagles

**DOI:** 10.3389/fmicb.2024.1437036

**Published:** 2024-09-17

**Authors:** Mengdi Zhao, Yueyao Li, Yuanyuan Zhang, Guangyu Li

**Affiliations:** ^1^College of Animal Science and Technology, Qingdao Agricultural University, Qingdao, China; ^2^College of Animal Science and Technology, Jilin Agricultural University, Changchun, China

**Keywords:** *Lactobacillus johnsonii*, beagle, whole genome sequencing, probiotic, functional food

## Abstract

Probiotics are one of the management tools to improve the host’s healthy microbiota. The positive effects of probiotics on host health are species-specific, so probiotics isolated from host’s own gut may be most beneficial. Many of the metabolites (e.g., short-chain fatty acids, bacteriocins, and hydrogen peroxide) produced by *Lactobacillus johnsonii* have specific inhibitory profiles against invading pathogens. In this study, we isolated *L. johnsonii* GJ231 from the intestinal tract of healthy female beagles. The genome size of 1.763 M encoded a total of 1,691 predicted genes. Many carbohydrate-active enzymes responsible for carbohydrate degradation and the production of short-chain fatty acids were also predicted. The metabolic profile of short-chain fatty acids in *L. johnsonii* GJ231 was determined using LC–MS/MS. The bacteriocin-producing gene bacteriocin (lactacin F) in *L. johnsonii* GJ231 was also predicted. *In vitro*, experiments demonstrated that GJ231 can thrive in weak acids, 0.3% bile salts, and artificial gastrointestinal fluid models. It was tolerant of to high temperatures up to 70°C, was non- hemolytic, inhibited pathogenic bacteria, and had a high antioxidant capacity. *In vivo* safety experiments conducted in mice revealed that oral administration of GJ231 not only had no toxic side effect but also increased their antioxidant capacity. In conclusion, combining the above test results, which collectively demonstrate that canine-derived *L. johnsonii* GJ231 was a non-pathogenic, acid-tolerant and bile-salt-tolerant probiotic strain that inhibits pathogenic bacteria and improves host antioxidant function. This may make it a promising candidate for the development of innovative functional foods for pets.

## 1 Introduction

Dogs and cats have been companions to humans for thousands of years (Grześkowiak et al., 2015). Nowadays, pet owners consider their pets as family members, so the health of pets is a major concern (Redfern et al., 2017). The health of companion animals depends on their gut microbiota (Grześkowiak et al., 2015). Pet owners and veterinarians have typically used antibiotics to treat or prevent animal diseases. However, the use of antibiotics can lead to gut microbiota disruption, and overuse can lead to a rapid increase in antibiotic residue and resistance (Ma et al., 2021; Shao et al., 2021). Close contact between pets and their owners can lead to the transfer of bacterial resistance, resulting in harm to both parties as a result (Scarborough et al., 2021; El-Razik et al., 2023). Therefore, there is a considerable need to develop new products to replace antibiotics.

In recent years, there has been a growing interest in the use of probiotics as an alternative to antibiotics for the prevention and treatment of bacterial infections, in particular *Lactobacillus* and *Bidobacterium* (Wan et al., 2019). Probiotics are “l*ive microorganisms that, when administered in adequate amounts, confer a health benefit on the host*” (Hill et al., 2014). Probiotics such as lactic acid bacteria (LAB) can inhibit the adhesion and activity of pathogenic bacteria, modulate immunity, improve intestinal barrier integrity, and produce organic acids as well as antimicrobial compounds (La Fata et al., 2018; Sanders et al., 2019). *Lactobacillus johnsonii*, a type of LAB, has been shown to improve antioxidant capacity, alleviate colitis, and improve hypercholesterolemia (Kim et al., 2006; Jia et al., 2022; Yoon et al., 2023).

Probiotic candidates isolated from host’s own gut may be most effective, as the co-evolution of gut microbes with the host can better prepare the strain to colonize and thrive in the host’s gastrointestinal tract (Garcia-Gutierrez et al., 2019; Johnson et al., 2023). A previous study reported that *L. johnsonii* strains were specific to their hosts (Buhnik-Rosenblau et al., 2012). Whole-genome sequencing, aided by the rapid advancement of sequencing technology, is becoming essential for studying microorganisms, presenting a new technique for investigating the probiotic potential and possible pathogenic features of microbes (Tyagi et al., 2022). In this study, we isolated *L. johnsonii* strain GJ231 from the gastrointestinal tract of healthy beagles. A comprehensive analysis of the probiotic potential and safety of strain GJ231 was conducted using whole-genome sequencing, along with *in vivo and in vitro* tests, demonstrating the strain’s potential for use in companion animals.

## 2 Materials and methods

### 2.1 Sample collection

Animals were acquired from the Jimo Pet Base of Qingdao Agricultural University. Six well-grown and healthy 6-month-old female beagles with an average weight of 5.45 ± 0.36 kg were selected. All test animals were not given probiotics or antibiotics in the past 2 months. A cotton swab was dipped into a small amount of saline and used for sampling. Samples were promptly transported to the laboratory.

### 2.2 Strain isolation and identification

Experimental samples were subjected to gradient dilution (10^−3^–10^−7^), and 50 μL of each dilution was coated onto Man, Rogosa, and Sharpe (MRS) agar plates (Haibo, China). The plates were incubated inverted at 37°C for 48–72 h. Single colonies with lysogenic rings were selected and transferred to MRS broth. After 2*–*3 passages, glycerol (25.0%) was added as a preservative, and cultures were stored for future use. Bacterial DNA was extracted with a Bacterial Genome Kit (Tiangen, China). Subsequently, polymerase chain reaction (PCR) was performed using the primers 16S-27F (5’-AGAGTTTGATCCTGGCTCAG-3′) and 16S-1492R (5’-TACGGCTACCTTGTTACGACTT-3′) (Wang et al., 2018) and the resulting products were sent to Qingke Biotech (Beijing, China) for sequencing. The sequencing results were compared with other sequence data at National Center for Biotechnology Information, and a phylogenetic tree (Neighbor Joining) was reconstructed using Mega 7.0 (Sudhir Kumar, King Abdulaziz University, Saudi Arabia) (Kumar et al., 2016).

*L. johnsonii* GJ231 used in this study has been conserved at the China Center for Type Culture Collection (CCTCC M2023981).

### 2.3 Probiotic potential

#### 2.3.1 Growth and acid production curves of strains

GJ231 cultured for 18*–*20 h was inoculated into MRS broth at 37°C with an inoculum amount of 2.5% (v/v) and then incubated for 0, 1, 2, 3, 6, 9, 12, 15, 18, 21, 24, 27, 30, 36, or 48 h, respectively. The optical density at 600 nm (OD_600_) was measured at each time point, while the pH value was determined at different time points using a pH detector (Shanghai INESA, China).

#### 2.3.2 Antimicrobial ability

Cultures in the exhibiting stationary phase after 18 h of incubation (1 × 10^8^ CFU/mL) and the bacterial suspension (BS) was centrifuged at 4°C, 10,621 × g (10,000 rpm in Centrifuge 5430R) for 10 min. The cell-free supernatant (CFS) was collected, and the remaining bacterial pellet (BP) was resuspended in the same volume of PBS. Similarly, cultures of pathogenic bacteria (*Escherichia coli* ATCC 25922; *Staphylococcus aureus* ATCC 25923; *Salmonella enterica* subsp. *Enterica* serovar Typhimurium ATCC 14028; *Pseudomonas aeruginosa* ATCC 27853; *Listeria monocytogenes* ATCC 19115) in the stationary phase of growth were adjusted to a concentration approximately 1 × 10^7^ CFU/mL. A two-layer method was adopted using Oxford cups on Tryptone Soy Agar (TSA) (Solarbio, China), with 100 μL of CFS, BS, BP, or CFS_7.0_ (CFS was adjusted to pH 7.0) added in each well. The wells were then incubated at 37°C for 24 h, and the inhibition zone diameter (IZD) was determined.

#### 2.3.3 High temperature tolerance

The method described by Zhao et al. (2023) was followed with minor modifications. Briefly, GJ231 bacterial solution cultured for 18–20 h was placed into a water bath heated to 37, 50, 60, 70, or 80°C for 5 min, respectively. Then was quickly transferred to ice for 30–60 s. The number of bacteria in 100 μL of the solution was then calculated by plate counting. The survival rate was calculated using the following formula:


(1) $$\mathrm{Survivability}\;\left(\%\right)=T{\mathrm{treatment}}^{1}/T{\mathrm{initial}}^{1}\times 100\%$$

Where *T*_initial_^1^ and *T*_treatment_^1^ are the number of bacteria (log CFU/mL) before and after treatment, respectively.

#### 2.3.4 Resistance to gastrointestinal environmental

The method described by Gilliland and Walker (1990) was followed with minor modifications. Briefly, GJ231 bacterial suspension cultured for 18–20 h were centrifuged at 4°C, 10,621 × *g* for 10 min and the supernatant was discarded. The bacterial cells were washed three times with PBS (pH 7.0). The cells were then resuspended in 0.1 and 0.3% (w/v) bile salt solutions, and the 0, 1, 2, and 4 h incubations were taken for counting using dilution plate counting method. Similarly, bacterial cultures from GJ231 were cultured until reaching the stationary phase, and samples were taken at 1.5 h or 2 h intervals after resuspension in gastric or intestinal fluids. The remaining steps were the same as those for pre-bile salt tolerance treatment. For the treatment with gastrointestinal fluid, samples were initially resuspended in gastric fluid for 1.5 h, followed by resuspension in intestinal fluid for 2 h before being sampled and counted. All cultures were incubated at 37°C, and survival rates were calculated using Equation 1.

#### 2.3.5 Antioxidant test

Ascorbic acid was used as a positive reference and a DPPH free radical scavenging capacity assay kit (Solarbio, China) was operated. The absorbance was measured at 515 nm (Cheng et al., 2021). Calculated using Equation 2.


(2) $$\begin{array}{l} \mathrm{scavenging}\;\mathrm{activity}\%\\ =\left[\frac{\left[{A_{\mathrm{blank}}}^{1}-\;\left({A_{\mathrm{sample}}}^{1}-{A_{\mathrm{control}}}^{1}\right)\right]}{{A_{\mathrm{blank}}}^{1}}\right]\times 100\% \end{array}$$

(2) A_sample_^1^ is the absorbance of the sample; A_control_^1^ is the absorbance of the mixture to sample and anhydrous ethanol; A_blank_^1^ is the absorbance of the mixture of the extract solution and working solution.

A working solution was made by mixing 7 mM ABTS and 2.45 mM potassium persulfate and after incubation for 12 h away from light. Add 20 μL of sample to 200 μL of working solution and mix well. Next, the reaction was carried out for 20 min at room temperature and protected from light, and the absorbance was measured at 734 nm. As a positive control, ascorbic acid was utilized. Calculated using Equation 3.


(3) $$\mathrm{scavenging}\;\mathrm{activity}\%=\left(1-\frac{{A_{\mathrm{sample}}}^{2}-{A_{\mathrm{control}}}^{2}}{{A_{\mathrm{blank}}}^{2}}\right)\times 100\%$$

(3) A_sample_^2^ is the absorbance of a mixture of sample and working solution; A_control_^2^ is the absorbance of the mixture of deionized water and sample; A_blank_^2^ is the absorbance of the mixture of deionized water and working solution.

O2- scavenging capacity was determined using the Superoxide Anion Scavenging Capacity Kit (Solarbio, China). The OD value was measured at 560 nm. Calculated using Equation 4 (Zhao et al., 2023).


(4) $$\mathrm{scavenging}\;\mathrm{activity}\;\%=\left(\frac{{A_{\mathrm{blank}}}^{3}-{A_{\mathrm{sample}}}^{3}}{{A_{\mathrm{blank}}}^{3}}\right)\times 100\%$$

(4) A_sample_^3^ is the absorbance of a mixture of sample and working solution; A_blank_^3^ is the absorbance of the mixture of deionized water and working solution.

#### 2.3.6 Autoaggregation activity

Cultures during the stationary phase were washed three times with PBS (pH 7.0), and the OD_600_ (*A*_0_) of the bacterial solution was determined. The bacterial solution was vortexed for 25*–*30 s and then incubated at 37°C for 8 h. The OD_600_ (*A*_1_) was then determined. Calculated using Equation 5.


(5) $$\mathrm{Autoaggregation}\;\mathrm{activity}\;\left(AAG,\%\right)=\left(1-A_{1}/A_{0}\right)\times 100\%$$

#### 2.3.7 Cell surface hydrophobicity

GJ231 was cultured at 37°C for 18–20 h and then centrifuged at 4°C at 10,621 × *g* for 10 min. Cells were washed three times with PBS (pH 7.0) and resuspended until the OD_600_ was approximately 0.25 ± 0.05 (*A*_2_). An equal volume of xylene and chloroform (1,1, v/v) was then added, and samples were vortexed for 120 s before incubating at 37°C for 3 h. The OD_600_ of the aqueous phase was then measured (*A*_3_). Calculated using Equation 6.


(6) $$\mathrm{Cell}\;\mathrm{Surface}\;\mathrm{Hydrophobicity}\;\left(CSH,\%\right)=\left(1-A_{3}/A_{2}\right)\times 100\%$$

#### 2.3.8 Hemolytic activity

A culture of GJ231 was streaked on the blood agar base (Oxoid, Germany) containing 5.0% (v/v) sheep blood, which were incubated at 37°C for 20–24 h (Nataraj et al., 2023). Blank blood plates without inoculation were used as a negative control (data not shown), and *Staphylococcus aureus* (ATCC 25923) was used as a positive control.

### 2.4 Complete genome sequencing analysis

GJ231 bacterial pellet and genomic DNA were extracted using the SDS extraction method (Wang et al., 2023c). Libraries were prepared using an SQK-LSK110 kit (Oxford, United Kingdom) following the manufacturer’s instruction manual using 1.0 g DNA. After sample purification and quality control, small fragment libraries were generated using a Universal Plus DNA Library Prep Kit for MGI V2 (Vazyme, China). The libraries underwent quality checks and were sequenced using the Nanopore PromethION and Illumina NovaSeq 6,000 platforms. Reads were assembled using Unicycler (Version: 0.5.0) software, plasmids were identified using PlasFlow software, and the assembled genome was used for coding gene prediction using Prokka (Version: 1.14.6) software (Seemann, 2014). Functional elements, genomic functions, database annotation, and websites were listed in Supplementary Table S1. Thresholds for carbohydrase annotation were set at an *E*-value of <1e-18 and coverage of >35.0%. Pathogen–host interaction thresholds were set at an E-value of <1e-5, and virulence factor annotation was performed using the VFDB database with a coverage of >40.0% (Wang et al., 2023b).

Potential gene clusters for natural product biosynthesis in GJ231 were predicted using the antiSMASH database. In addition, the bacteriocin production gene cluster was predicted using the Bagel4 database. The amino acid sequences of bacteriocin lactacin F (*lafA* and *lafx*), which have the highest sequence similarity, were submitted to the SWISS-MODEL web server to generate protein models (Wang et al., 2024).

### 2.5 Safety evaluation *in vivo*

Forty Kunming white mice (from Jinan Pengyue Experimental Animal Breeding Co., Ltd.) were randomly divided into two groups, with an equal number of males and females in each group. The oral administration experiment began after 7 d of acclimatization with unrestricted access to food and water, and a room temperature of 25°C. The control group (CK) was administered 0.1 mL of saline daily via oral gavage for 28 d, while the GJ231 group was administered an equal volume of GJ231 bacterial solution (1 × 10^9^ CFU/mL) daily via oral gavage for 28 d. Throughout the study, mice were monitored daily for diarrhea, and their body weights and food intake were recorded weekly. Before the end of the experiment, the mice were fasted for 12 h, rendered unconscious with 1.0% (w/v) sodium pentobarbital (50 mg/kg), and blood was collected from the aorta. Blood samples were centrifuged at 825 × *g* (3,000 rpm, using a centrifuge 5,702 Eppendorf, Germany) at 4°C for 15 min. The serum was collected and assayed for biochemical markers using a kit (Nanjing, China). Subsequently, the hearts, livers, spleens, kidneys, and thymuses of the mice were observed and collected. The organs of 10 mice in each group were randomly selected and added to 10 mL of sterile saline (1.0 g/mL). The organs were homogenized and crushed, and 0.1 mL of the homogenate was spread evenly on MRS plates. The plates were then incubated at 37°C for 48 h for observation.

### 2.6 Short chain fatty acids

Short-chain fatty acids in the supernatant of strain GJ231 were determined using LC–MS/MS. Briefly, 30 to 40 mg of frozen faecal samples were placed into a 1.5 mL centrifuge tube. Then, 1 mL of 50% acetonitrile (ACN, Fisher Chemical, United States) was added, followed by 2 to 3 metal grinding beads. The sample was processed in an E6618 tissue grinder (Beyotime, China) for 1 min at 60 Hz and then centrifuged at 15871 × *g* (13,000 rpm in a Centrifuge 5,430 Eppendorf, Germany) for 10 min at 4°C. One hundred microliters of supernatant was taken and diluted proportionally to 10 mg of sample per 1.8 mL of 50% ACN solution. The mixture was vortexed and shaken (Scilogex, USA) for 30 s and then centrifuged at 15871 × *g* for 30 s. Twenty microliters of the supernatant was aspirated, and 10 μL of 200 mM 3-nitrophenylhydrazine-HCl (3-NPH-HCl, Sigma Aldrich, United States) was added separately. Ten microlitres of 200 mM N-(3-dimethylaminopropyl)-N′-ethylcarbodiimide-HCl (EDC-HCl, Sigma Aldrich, United States), 80 μL of 50% ACN, 50 μL of 7% pyridine (Sigma–Aldrich, United States), and 1 μL of isotope internal standard solution (Toronto Research Chemicals, Canada) were vortexed and shaken for 3 min, derivatized in a constant-temperature water bath at 40°C for 30 min and centrifuged at 15871 × *g* for 1 min at 4°C. Finally, 20 μL of the reaction solution after derivatization was added to 280 μL of 50% ACN, vortexed for 30 s, centrifuged at 15871 × g for 10 min at 4°C, aspirated into the injection vial, and then subjected to LC–MS/MS analysis (LC-30 HPLC, SCIEX QTRAP 5500 mass spectrometry, Phenomenex: Kinetex C18, 2.6 μm 100 × 3.00 mm, column temperature: 40°C, flow rate: 0.7 mL/min).

### 2.7 Statistical analysis

Data were expressed as the mean ± standard error of mean (SEM). Statistical significance was determined using t-tests and one-way analysis of variance in GraphPad Prism 8.3.0, with statistical significance set at a level of *p* < 0.05.

### 2.8 Data availability

The whole-genome sequence data has been deposited under accession number PRJNA1028141 (https://www.ncbi.nlm.nih.gov/bioproject/PRJNA1028141) (17-Oct-2023).

## 3 Results

### 3.1 Identification of GJ231

**Figure 1:**
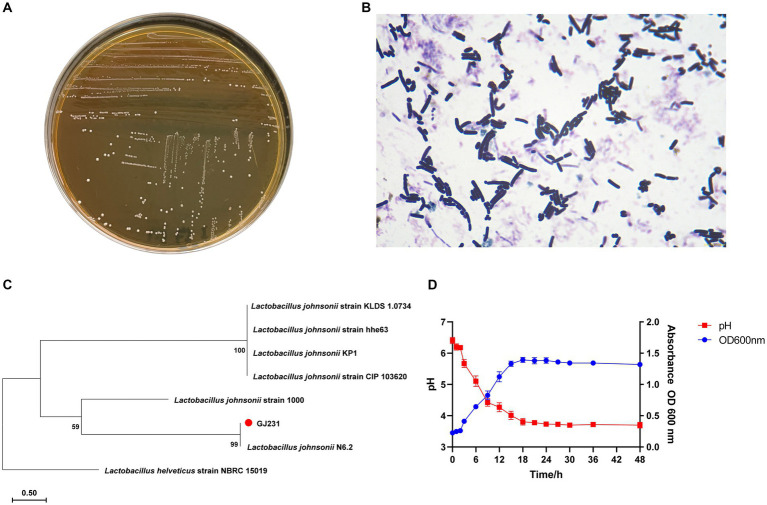
Morphology, staining and phylogenetic tree of strain GJ231. **(A)** Morphology of strain GJ231, **(B)** gram staining results of strain GJ231, **(C)** neighbor-joining phylogenetic tree of strain GJ231 (*Lactobacillus helveticus* as an exogenous species) and **(D)** growth and acid-producing curve.

The results of physiological and biochemical experiments on strain GJ231 were shown in Table 1. Strain GJ231 could utilize glucose, galactose, maltose, fructose, raffinose, rhamnose, xylose and sucrose. The colony morphology of strain GJ231 on MRS agar plates was raised, creamy white, and spherical or ovoid (Figure 1A). After Gram staining, bacteria appeared under a microscope as purple, rod-shaped cells with rounded ends, indicating that they are Gram-positive (Figure 1B). By reconstructing an evolutionary tree, we determined that GJ231 shared a nucleotide similarity of 99% with *L. johnsonii* N6-2 (Figure 1C). We therefore concluded that GJ231 represented *L. johnsonii*.

**Table 1:** Physiological and biochemical characteristics of *Lactobacillus johnsonii* GJ231.

Items	Results ^a^	Items	Results^a^	Items	Results^a^
Aesculin	−	Mannitol	−	Salicin	−
Glucose	+	Sorbitol	−	Rhamnose	+
Cellobiose	−	Raffinose	+	Xylose	+
Galactose	+	Hydrogen sulfide	−	Mobility	−
Maltose	+	1% sodium equine	−	Sucrose	+
Fructose	+	Methyl Red test	−	Inulin	−
L-arabinose	−	Voges-Prokauer test	−	Gelaune liquefaction	−

^a^+: positive; −: negative.

### 3.2 Growth and acid production curve

*L. johnsonii* GJ231 was in the lag period from 0 to 3 h, entered the exponential phase of growth after 3 h and reached the stationary phase at 18 h. In addition, the strain had a good capacity for acid production (Figure 1D).

### 3.3 Antimicrobial ability

**Figure 2:**
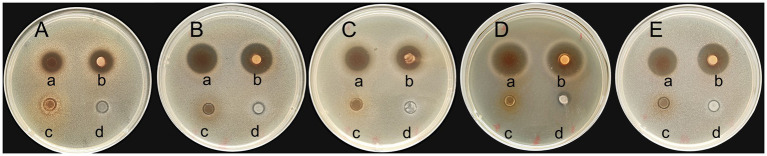
The inhibitory effects of GJ231 against pathogenic indicator bacteria. **(A)** *Escherichia coli*; **(B)** *Staphylococcus aureus*; **(C)** *Salmonella*; **(D)** *Listeria monocytogenes*; and **(E)** *Pseudomonas aeruginosa*. In each TSA agar, (a) added the cell-free supernatant of GJ231; (b) added the bacterial suspension of GJ231; (c) added the cell-free supernatant (pH = 7.0) of GJ231; (d) added the bacterial pellet.

The bacteriostatic activity of GJ231 against five pathogenic bacteria (Figure 2; Supplementary Table S2) was observed, with the most significant bacteriostatic effect observed against *L. monocytogenes*, followed by *P. aeruginosa*, *S. aureus*, *Salmonella*, and *E. coli*. However, there was no significant difference in the inhibitory effects of BS or CFS against the same pathogenic bacteria (*p* > 0.05). When the pH of the CFS was adjusted to pH 7.0, the inhibitory effect disappeared, suggesting that organic acids may play the primary inhibitory role in the CFS of GJ231. Furthermore, the BP of GJ231 had no inhibitory effect on pathogenic bacteria.

### 3.4 Complete genome sequencing and bioinformatic analysis

**Figure 3:**
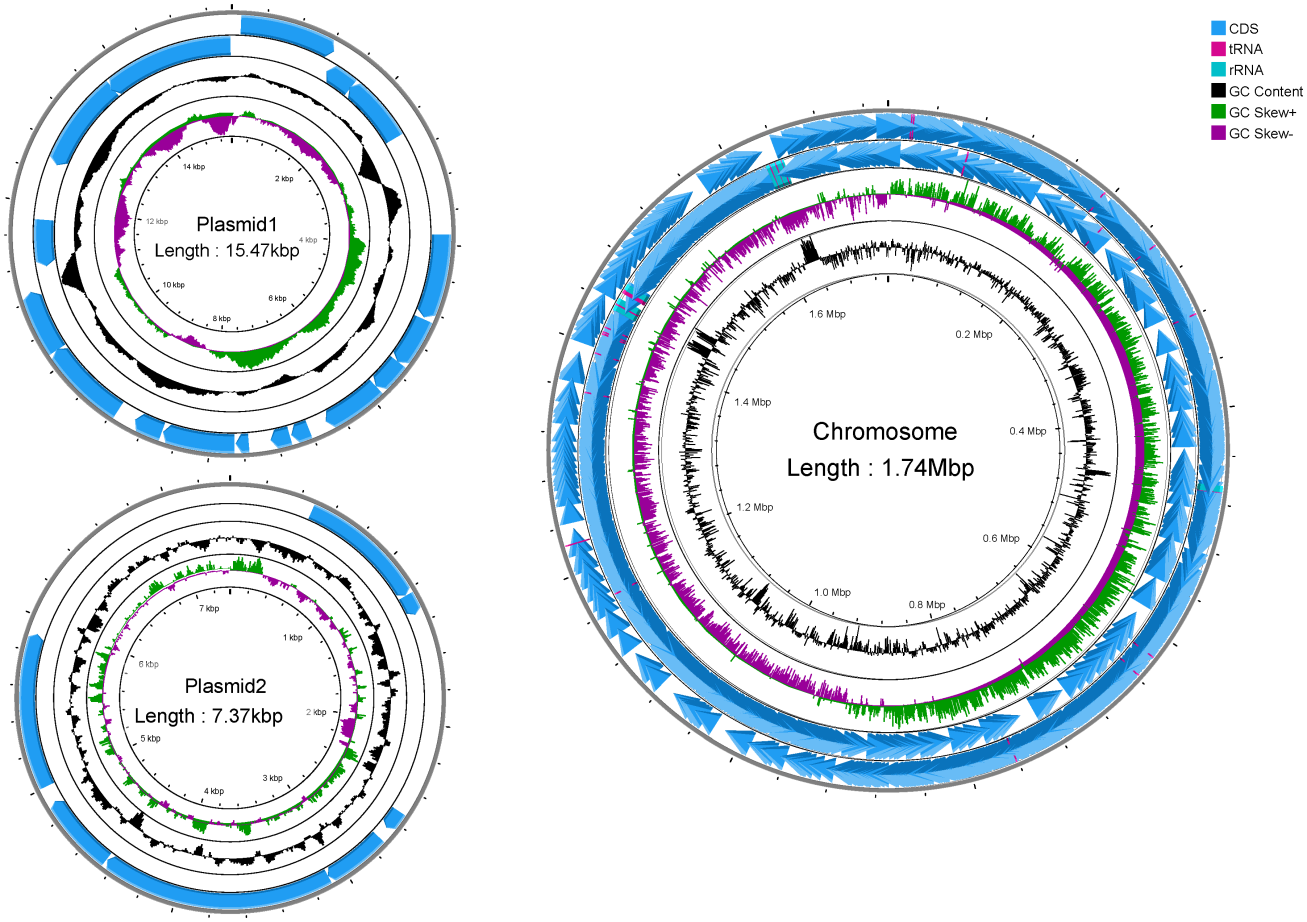
Complete genome map of *Lactobacillus johnsonii* GJ231.

Genome-wide sequencing revealed that GJ231 contained one chromosome with a length of 1,740,019 bp and two plasmids; these had a G + C content of 34.70, 35.73, and 34.32%, respectively (Table 2; Figure 3). There were 1,691 coding sequences (CDSs) with a total length of 1,553,460 bp. Furthermore, we identified 152 pseudogenes, 7 rRNAs, and 78 tRNAs. Our analysis revealed three genomic islands (Supplementary Table S3), eight prophages (Supplementary Table S4), 189 repeated sequences totaling 17,652 bp in length (Supplementary Table S5), 17 insertions (Supplementary Table S6), two cytochromes P450 (Supplementary Table S7), and 83 virulence factors (Supplementary Table S8). In addition, there were 49 carbohydrate-active enzymes, with 26 being glycoside hydrolases and many being glycosyltransferases (Table 3). Moreover, the whole genome of GJ231 contained a 185-bp CRISPR-Cas sequence located on the chromosome (Supplementary Table S9). In addition, three resistance genes, namely *InuA*, *ErmT*, and *ErmB*, were predicted (Table 4).

**Table 2:** General genome features of *Lactobacillus johnsonii* GJ231.

Indicator	Number or content
Chromosome (bp)	1,740,019
Plasmid1 (bp)	7,371
Plasmid2 (bp)	15,479
G + C content of chromosome (%)	34.70%
G + C content of plasmid1 (%)	35.73%
G + C content of plasmid2 (%)	34.32%
Coding gene numbers	1, 691
Total length of coding genes (bp)	1,553,460
Pesudogene size (bp)	66,354
Pesudogene number	152
rRNAs (16 S–23 S-5S)	7
tRNA	78
tmRNA	1
misc_rna	34

**Table 3:** CAZymes-encoding genes of *Lactobacillus johnsonii* GJ231.

CAZymes class	Gene counts
Glycoside Hydrolases (GHs)	26
Glycosy Transferases (GTs)	21
Carbohydrate Esterases (CEs)	1
Carbohydrate-Binding Modules (CBMs)	1
Total	49

**Table 4:** Resistance gene prediction of *Lactobacillus johnsonii* GJ231.

Class	Resistance gene	Identity	Alignment length/Gene length	Phenotype
Lincosamide	*Inu(A)*	99.38	100	Lincomycin
Lincosamide Aminoglycosides Macrolide	*Erm(T)*	99.17	98	Lincosamide, Streptogramin_b, Macrolide
	*Erm(B)*	99.18	100	

**Figure 4:**
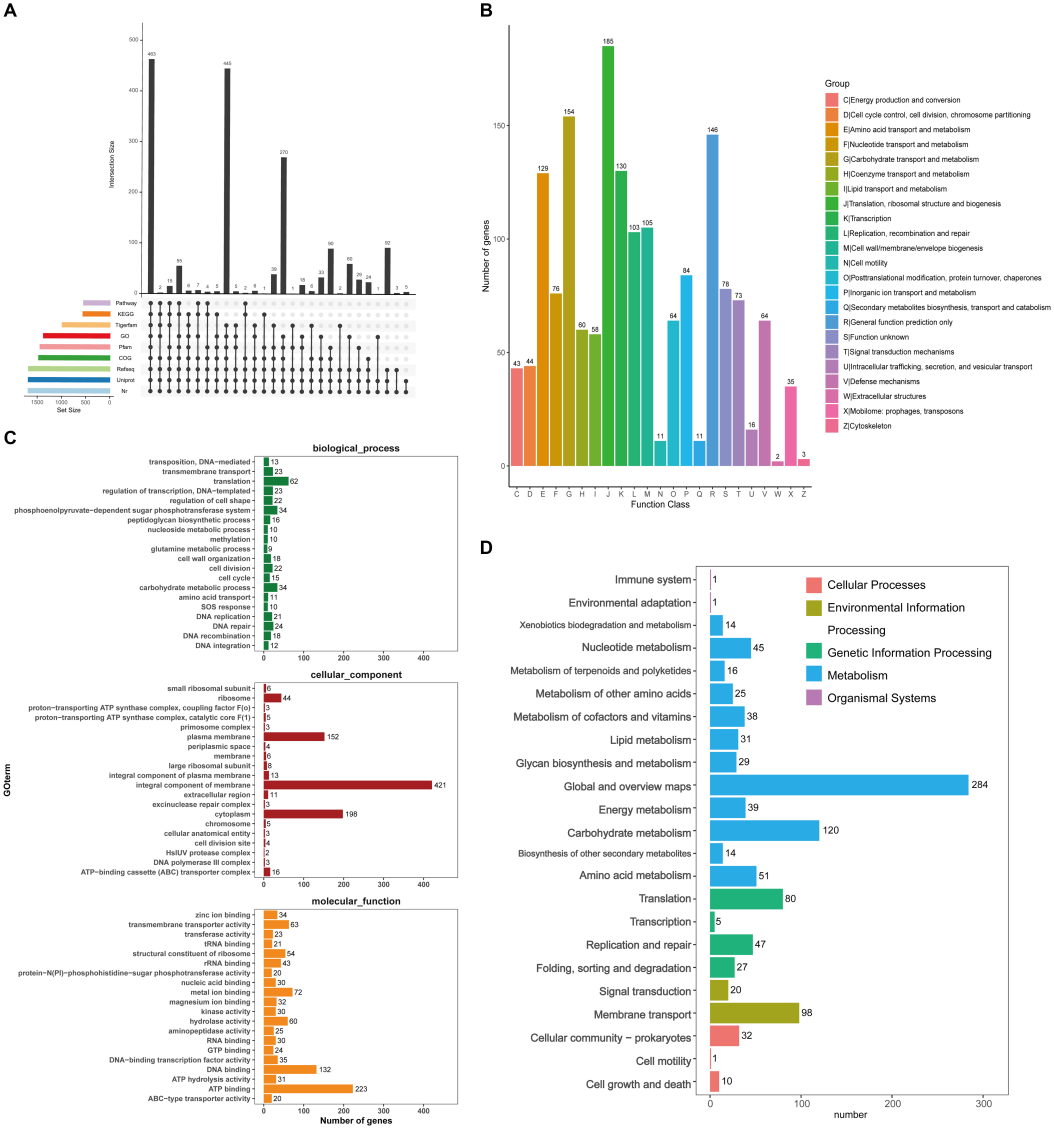
Prediction function of *Lactobacillus johnsonii* GJ231. **(A)** Coding gene database annotation statistics; **(B)** COG; **(C)** GO analysis and **(D)** KEGG pathways of proteins functional.

Figure 4A showed a map of the coding genes in GJ231 annotated in the universal database. A total of 1,674 genes were predicted using the COG database (Figure 4B). Translation and carbohydrate metabolic processes, as well as the phosphoenolpyruvate-dependent glucose phosphotransferase system, were identified as highly active biological processes. We also determined that the plasma membrane, an essential cellular component, plays a crucial role in cellular function, while ATP binding, DNA binding, and metal ion binding were essential molecular functions (Figure 4C). KEGG annotation revealed 1,498 genes classified into 23 functional groups. These groups included global and overview maps (284 genes), carbohydrate metabolism (120 genes), and membrane transport (98 genes), as shown in Figure 4D. The database annotation results indicated that *L. johnsonii* GJ231 has a robust metabolic capacity and can adapt to multiple ecological niches.

**Figure 5:**
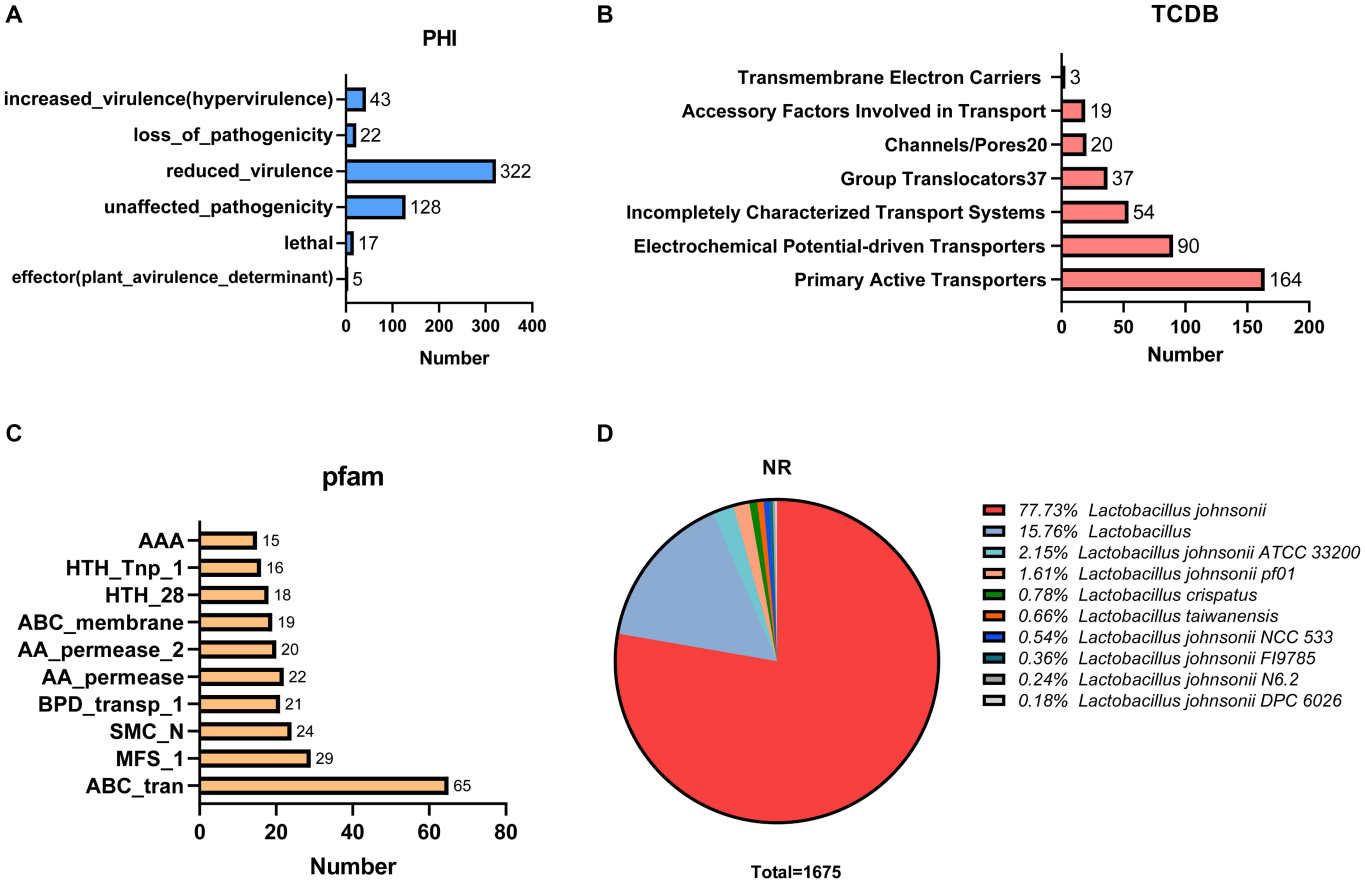
Proprietary database annotations of *Lactobacillus johnsonii GJ231*. **(A)** Pathogen host interactions annotations; **(B)** TCDB transporter protein; **(C)** PFAM domain and **(D)** NR annotations of *Lactobacillus johnsonii* GJ231.

We annotated 537 pathogen–host interactions (Figure 5A). Furthermore, we identified 387 membrane transporter proteins, with primary active transporters being the most numerous at 164 (Figure 5B). The three most common structural domains identified were 65 ABC transporters (ABC_tran), 29 Major Facilitator Superfamily (MFS_1) domains, and 24 RecF/RecN/SMC_N terminal domains (SMC_N) (Figure 5C). The amino acid sequence database of non-redundant (NR) proteins was also analyzed, consistent with the results of 16S rRNA gene sequencing (Figure 5D). In addition, genes conferring resistance to acid, bile salts, high temperatures, and oxidative stress were also identified in the genome of GJ231 (Table 5).

**Table 5:** Probiotic genes that render resistance to *Lactobacillus johnsonii* GJ231 in harsh gastric conditions.

Genes detected in GJ231	EC Number	Predicted function
Adenosine triphosphate (ATP) synthase F0 sector subunit a	EC 3.6.3.14	Acid tolerance
ATP synthase F0 sector subunit c	EC 3.6.3.14	
ATP synthase subunit alpha	EC 3.6.3.14	
ATP synthase subunit gamma	EC 3.6.3.14	
ATP synthase subunit beta	EC 3.6.3.14	
ATP synthase subunit epsilon	EC 3.6.3.14	
ATP synthase subunit delta	EC 3.6.3.14	
Na+/H+ antiporter NhaC		
Na+/H+ antiporter NhaD		
Na+/H+ antiporter		
Phosphotransferase system cellobiose-specific component IIA	EC 2.7.1.205	
Phosphotransferase system cellobiose-specific component IIB	EC 2.7.1.205	
Phosphotransferase system cellobiose-specific component IIC		
L-lactate dehydrogenase	EC 1.1.1.27	
L-lactate permease		
ATP-dependent Clp endopeptidase proteolytic subunit ClpP	EC 3.4.21.92	
Glucose-6-phosphate isomerase	EC 5.3.1.9	
Guanosine triphosphate (GTP) pyrophosphokinase	EC 2.7.6.5	
Pyruvate kinase	EC 2.7.1.40	
Glucosamine-6-phosphate deaminase	EC:3.5.99.6	Acid/Bile tolerance
Cytosine triphosphate (CTP) synthase	EC:6.3.4.2	
Manganese-dependent inorganic pyrophosphatase (*ppaC*)	EC:3.6.1.1	
Choloylglycine hydrolase *cbh*		
Asp23/Gls24 family envelope stress response protein		Stress response
universal stress protein		
peroxide stress protein YaaA		
RpiR family transcriptional regulator		Bile inducible genes
RpiR family transcriptional regulator YbbH		
Secondary bile acid biosynthesis		
Heat-inducible transcriptional repressor *HrcA*		Temperature
Hsp33 family molecular chaperone HslO		
*DnaJ*		
*DnaK* (hsp70)		
Heat shock protein HtpX		
Small heat shock protein IbpA (HSP20)		
Ribosomal 50S subunit-recycling heat shock protein		
Molecular chaperone GrpE (heat shock protein HSP-70)		
Cold shock protein, CspA family		
Cold shock domain-containing protein *CspD*		
DEAD/DEAH box helicase		
S-ribosylhomocysteine lyase luxS	EC:4.4.1.21	*Biofilm formation*
Catabolite control protein A *ccpA*		
Family DNA-binding protein ComEA		
DCMP deaminase *comEB*	EC:3.5.4.12	
DNA internalization-related competence protein *ComEC*		
Competence protein ComGC		
Biofilm regulatory protein A		
Biofilm formation stimulator VEG		
Regulator of purF expression and biofilm formation		
peroxide stress protein YaaA		Peroxide
alkyl hydroperoxide reductase subunit F	EC:1.8.1.7	
nickel-type superoxide dismutase maturation protease	EC:3.4.21.89	Superoxide
removal of superoxide radicals	EC:1.8.1.9	
Putative oxidoreductase		Hydroxyl radicals
NAD(P)/FAD-dependent oxidoreductase	EC:1.8.1.7	
FAD dependent oxidoreductase	EC:1.3.5.4	
SDR family NAD(P)-dependent oxidoreductase	EC:1.1.1.100	
Pyridine nucleotide-disulphide oxidoreductase	EC:1.8.1.7	
Guanosine 5′-monophosphate oxidoreductase	EC:1.7.1.7	
DsbA family oxidoreductase		
NADPH-dependent oxidoreductase		
Fatty acid repression mutant protein (predicted oxidoreductase)		
PPOX class F420-dependent oxidoreductase		
MocA family oxidoreductase		
Thiol peroxidase		Oxidative stress
Thioredoxin family protein		
Arsenate reductase (thioredoxin)		
Thioredoxin		
Thioredoxin-disulfide reductase	EC:1.8.1.7	
Hsp33 family molecular chaperone *HslO*		

**Figure 6:**
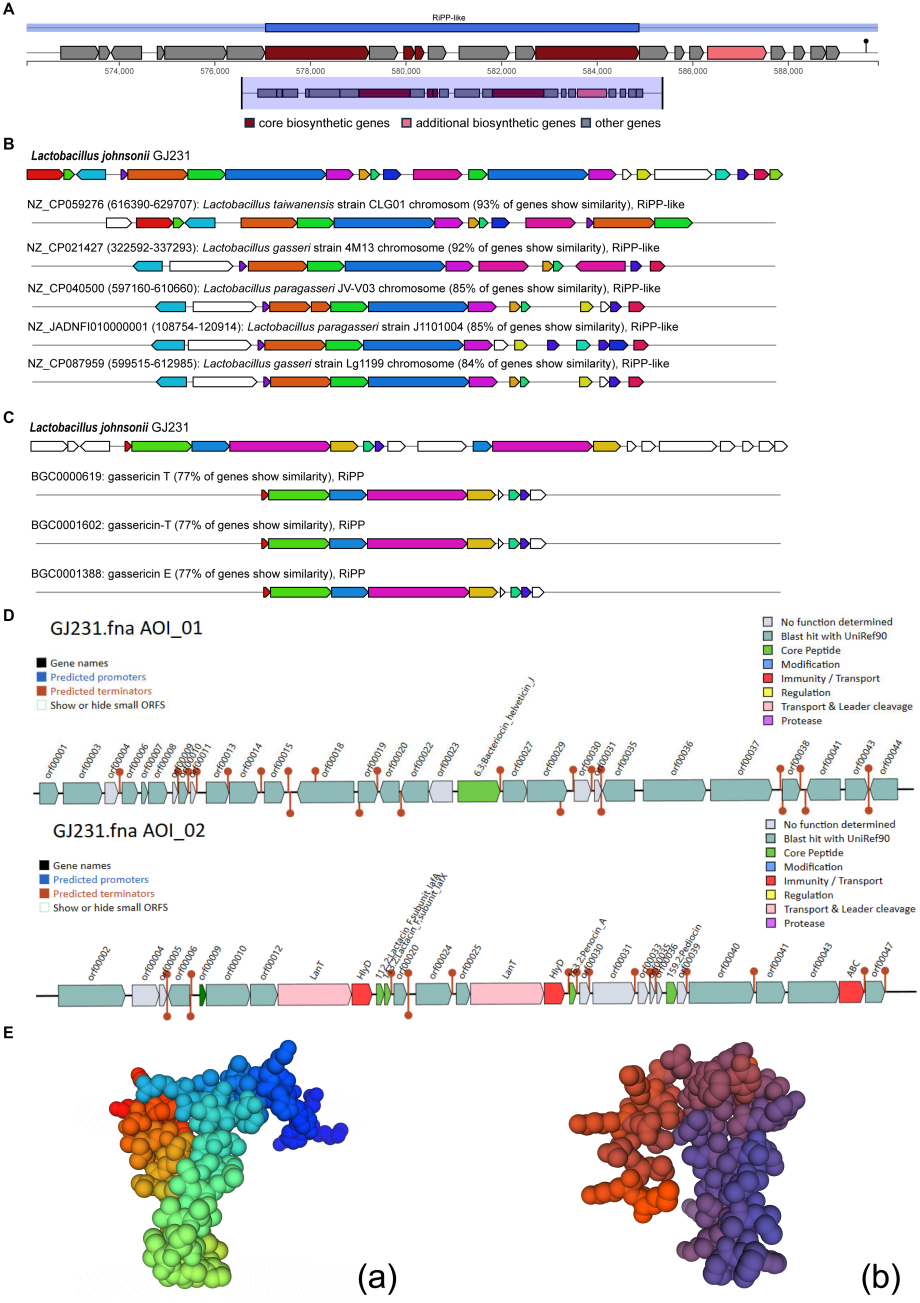
Bacteriocin prediction of *Lactobacillus johnsonii GJ231*. **(A)** Region of RiPP-like; **(B)** Cluster Blast; **(C)** Known Cluster Blast; **(D)** bacteriocin prediction by BAGEL 4 and **(E)** Protein tertiary structure prediction of *Lactobacillus johnsonii* GJ231.

We used the antiSMASH database to identify potential gene clusters for natural product biosynthesis in GJ231, and two potential products, gassericin E and gassericin T, were identified (Figures 6A–C). In addition, several potential bacteriocins were predicted in the GJ231 genome, such as gassericin, lactacin F (*lafA* and *lafx*), pediocin, and bacteriocin helveticin J (Figure 6D). Furthermore, the tertiary structure of the protein lactacin F (*lafA* and *lafx*) was modeled (Figure 6E).

### 3.5 Evaluation of the probiotic potential

**Figure 7:**
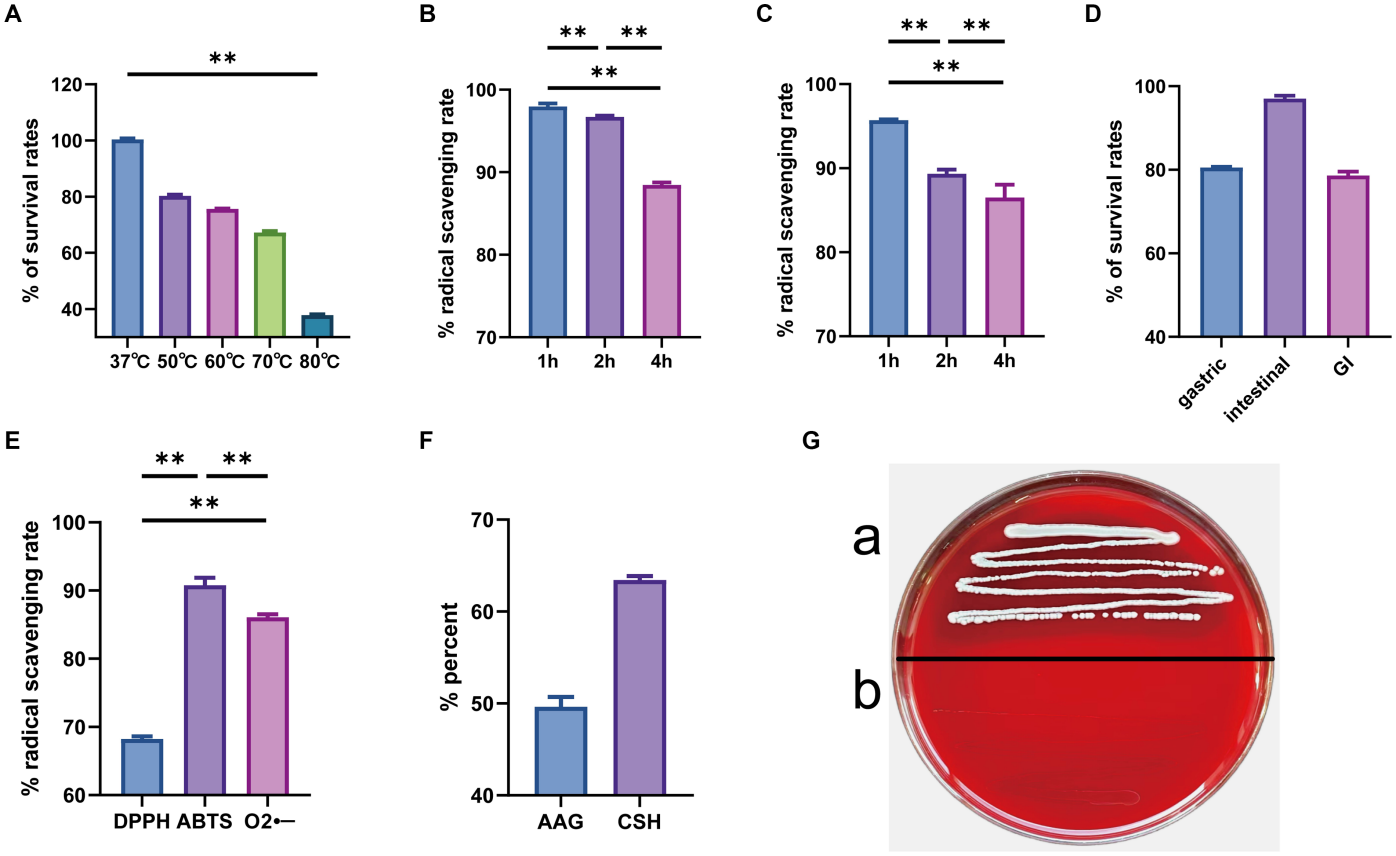
*In vitro* evaluation of probiotic potential. **(A)** High temperature resistance; **(B)** 0.1% bile salt resistance; **(C)** 0.3% bile salt resistance; **(D)** gastrointestinal tract models; **(E)** radical scavenging rate; **(F)** autoaggregation ability and cell surface hydrophobicity; **(G)** hemolysis tests (a. *Staphylococcus aureus* and b. GJ231). **p < 0.01 indicate differences between different types of treatment of GJ231.

Strain GJ231 tolerated high temperatures well, with over 60.0% survival after treatment at 70°C and approximately 40.0% survival after treatment at 80°C (Figure 7A). The survival rate was still greater than 85.0% with 0.1% bile salt treatment and greater than 80.0% with 0.3% bile salt treatment (Figures 7B,C). Moreover, the survival rate of strain GJ231 after treatment with gastric fluid was almost 80.0%, the attrition rate of strain GJ231 after treatment with intestinal fluid was very low, and the survival rate after co-treatment with gastrointestinal fluid was about 79.0% (Figure 7D). In addition, strain GJ231 exhibited significantly lower DPPH radical scavenging compared with ABTS and O_2_- scavenging (Figure 7E; *P* < 0.01). Adhesion of the strain is also an important indicator of *in vitro* probiotic properties. Strain GJ231 exhibited 50.0% autoaggregation activity and greater than 60.0% cell surface hydrophobicity (Figure 7F). Furthermore, GJ231 tested negative for hemolysis (Figure 7Gb), while *Staphylococcus aureus* exhibited β-hemolysis (Figure 7Gb). LC–MS/MS results showed that the metabolite (short-chain fatty acids) was mainly acetic acid at 1304.31 μg/mL (Table 6).

**Table 6:** Short-chain fatty acid content of the supernatant of *Lactobacillus johnsonii* GJ231.

Items	Content (ug/mL)
Acetic acid	1,385.187 ± 135.407
Propionic acid	4.904 ± 0.022
Isobutyric acid	1.674 ± 0.025
Butyric acid	0.775 ± 0.023
Isovaleric acid	1.146 ± 0.164
Valeric acid	0.059 ± 0.019
Caproic acid	0.268 ± 0.018
Enanthic acid	0.0854 ± 0.004
Caprylic acid	0.094 ± 0.017
Pelargonic acid	0.286 ± 0.024
Decanoic acid	0.036 ± 0.008

Values are mean with SD of three replications.

### 3.6 Safety evaluation *in vivo*

**Figure 8:**
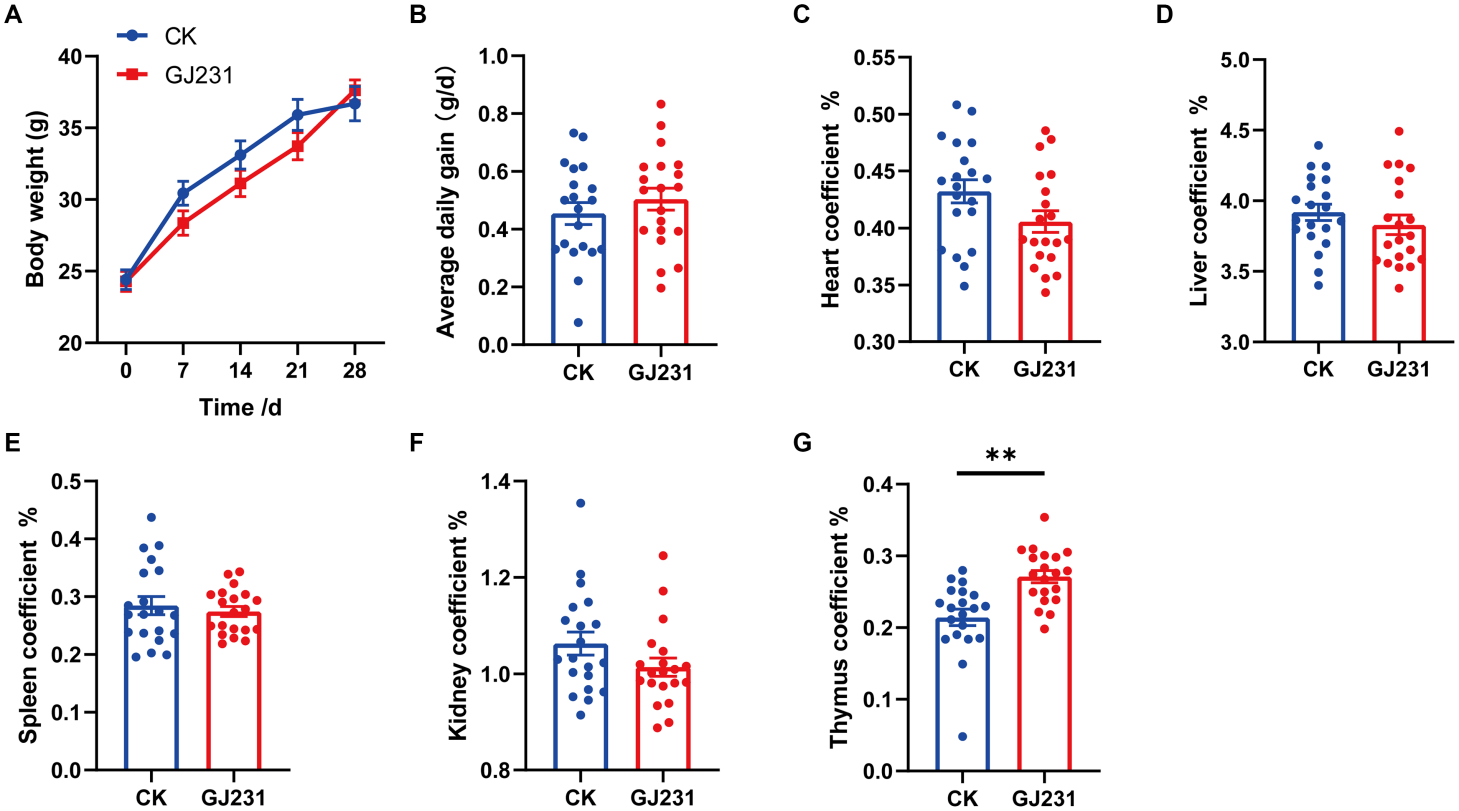
Effect of oral administration of *Lactobacillus johnsonii* GJ231 on organ indices in mice. **(A)** Body weight; **(B)** average daily gain; **(C)** heart coefficient; **(D)** liver coefficient; **(E)** spleen coefficient; **(F)** kidney coefficient and **(G)** thymus coefficient in mice. **p < 0.01 indicate differences between different types of treatment of *Lactobacillus johnsonii* GJ231, *n* = 20.

**Figure 9:**
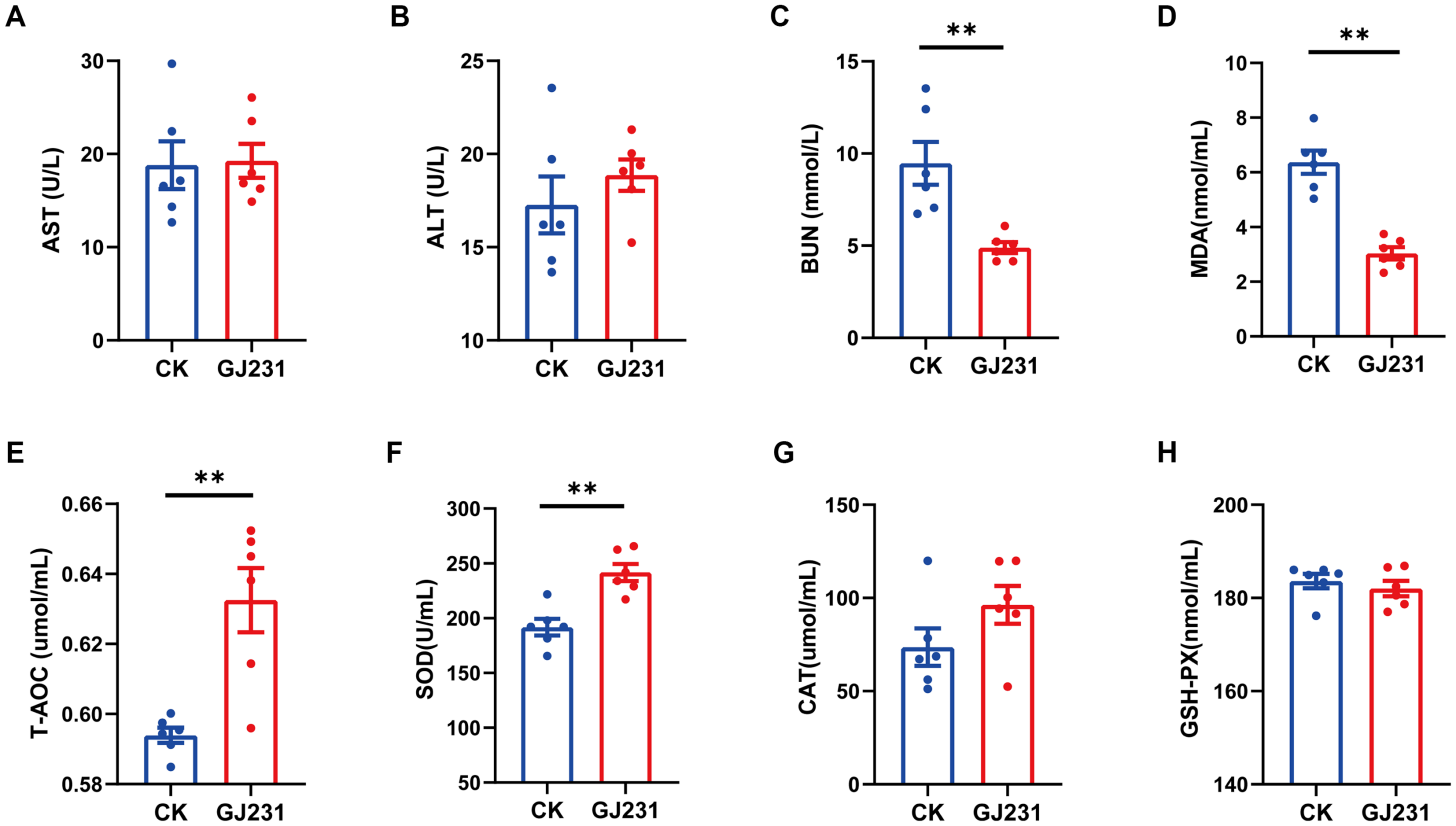
Effect of oral administration of *Lactobacillus johnsonii GJ231* on serum indices in mice. **(A)** Aspartate amino transferase; **(B)** alanine aminotransferase; **(C)** blood urea nitrogen; **(D)** malondialdehyde; **(E)** total antioxidant capacity; **(F)** superoxide dismutase; **(G)** catalase; and **(H)** glutathione peroxidase in mice. **p < 0.01 indicate differences between different types of treatment of *Lactobacillus johnsonii* GJ231, *n* = 6.

Safety experiments for the oral administration of GJ231 in mice were conducted. Throughout the oral experiment, all mice were active and did not develop diarrhea, die, or exhibit any other signs of illness. No significant pathological changes were observed in the various organs during autopsy. The body weight, average daily gain, heart coefficient, liver coefficient, spleen coefficient, and kidney coefficient of mice orally administered with GJ231 were not significantly different from those of the CK group (Figures 8A–F; *P* > 0.05). The thymus coefficient of mice orally administered with GJ231 was significantly higher than that of the CK group (Figure 8G; *P* < 0.01). The liver and kidney function indexes of mice were also measured. There was no significant difference in aspartate aminotransferase (AST) and alanine aminotransferase (ALT) levels in the oral GJ231 group compared with those in the CK group (Figures 9A,B; *P* > 0.05). However, the blood urea nitrogen (BUN) and malondialdehyde (MDA) contents were significantly lower than those in the CK group (Figures 9C,D; *P* < 0.01). In addition, the total antioxidant capacity (T-AOC) and superoxide dismutase (SOD) levels in mice orally administered with the GJ231 group were significantly higher than those in the CK group (Figures 9E,F; *P* < 0.01). However, the catalase (CAT) and Glutathione peroxidase (GSH-PX) levels did not show a significant difference compared with those in the CK group (Figures 9G,H; *P* > 0.05).

## 4 Discussion

Evaluating probiotic strains by combining phenotypic characterization and whole-genome sequencing analysis provides more comprehensive information about their potential biological properties than using one method of evaluation alone and is one of the most effective methods for exploring high-quality probiotic resources (Evanovich et al., 2019). In this study, a strain GJ231 from the healthy beagles was evaluated by a combination of whole genome sequencing and *in vitro* and *in vivo* experiments.

Whole-genome sequencing can analyze the complete genetic information of a strain at the gene level, providing a fast and accurate method for evaluating the function and safety of probiotics (Umanets et al., 2023). In this study, the whole-genome sequencing of strain GJ231 revealed that the strain had a genome size of 1.763 M with one chromosome and a G + C content of 34.70%, which was consistent with previous reports (Chen et al., 2023). Compared with *Lactiplantibacillus plantarum* and *Lactobacillus rhamnosus*, *L. johnsonii* has a smaller genome and lower G + C content, indicating a high degree of variation among different species of *Lactobacillus* to facilitate better adaptation to the environment (Jarocki et al., 2018). Understanding how carbohydrates are processed by the gut microbiota can help us investigate the influence of dietary carbohydrates on host health, as carbohydrate-active enzymes play a crucial role in host nutrient metabolism. The GJ231 genome contains 49 CAZymes: 26 glycoside hydrolase (GH, 53.06%) genes, 21 glycosyl transferase (GT, 42.86%) genes, one carbohydrate esterase (CE, 2.04%) gene, and one carbohydrate-binding module (CBM, 2.04%). It is worth noting that GJ231 contains numerous genes encoding GHs and GTs, which catalyze the transfer of sugars to specific receptors and play a crucial role in forming surface structures recognized by the host immune system. GHs catalyze the cleavage of glycosidic bonds, releasing abundant energy. Both GHs and GTs can help the host to resist the invasion and adhesion of potential pathogenic bacteria and their toxins to the intestinal epithelium (Mazmanian et al., 2008; Becerra et al., 2015). In addition, the presence of CRISPR-Cas was detected in the GJ231 genome, indicating that strain GJ231 may inhibit horizontal transfer of virulence or antibiotic resistance genes and has the capacity to resist foreign genetic elements (phage, plasmids, and insertion sequences) (Kim et al., 2021).

Transporter Classification Database (TCDB) is a database that categorizes membrane transport proteins (Saier et al., 2006). Transporter proteins are essential for bacterial life activities as they are involved in the uptake of nutrients and defense against endogenous and environmental stresses (Piepenbreier et al., 2017). The most prominent transporters in the GJ231 genome were Primary Active Transporters (164, 42.38%) followed by Electrochemical Potential-driven Transporters (90, 23.26%). Furthermore, the most PFAM-annotated proteins in strain GJ231 were ABC transporter proteins (65), which were involved in critical host transport processes and play critical roles in host defense and antibiotic resistance (Feng et al., 2020). The ABC transporter proteins in GJ231 may have similar functions. Moreover, when the GJ231 genome was annotated using the NR Database, the highest number of genes was annotated to *L. johnsonii*, consistent with the results of 16S rRNA gene sequencing.

The bacteriostatic effects of LAB have been widely reported (Adetoye et al., 2018; Mani-López et al., 2022). LAB can inhibit the growth of pathogenic bacteria through metabolites such as organic acids, extracellular polysaccharides, and bacteriocins. By decreasing intracellular pH and enabling the accumulation of ionized forms of organic acids in the cytoplasm, organic acids, especially lactic and acetic acids, exercise bactericidal action (van Zyl et al., 2020). The results of this study showed that a BS of GJ231 and the CFS were bacteriostatic against five pathogenic bacteria. We hypothesized that the organic acids were responsible for bacteriostatic activity of the CFS, as the bacteriostatic effect disappeared when the pH was changed. Therefore, we examined the organic acid content in the supernatant of strain GJ231 and the results showed a high content of acetic acid. It was noteworthy that the GJ231 genome also contained clusters of secondary metabolic genes, such as gassericin E and gassericin T, as well as gene encoding bacteriocins including gassericin, lactacin F, pediocin, and bacteriocin helveticin J. Chen et al. (2023) predicted that bacterioncin helveticin J was present in every bacteriocin-containing strain of *L. johnsonii*. Lactacin F, a two-component class II peptide bacteriocin, has been reported to reduce inflammation in patients with inflammatory bowel disease (Allison et al., 1994; Miri et al., 2022). Bacteriocin is one of the critical factors in the ability of probiotics to inhibit pathogens in the gastrointestinal tract (GIT). The antimicrobial activity of bacteriocins provides bacteriocin-producing probiotic strains with a competitive advantage in the complex GIT environment. These exciting phenomena will be studied more in depth in future research.

Probiotics are widely used in pets to prevent or combat diseases as they improve overall health. With the increasing variety of probiotic strains, the safety evaluation of probiotics has become a popular issue (Ishibashi and Yamazaki, 2001; Wang et al., 2023a). Hemolytic strains may cause sepsis in the host. Therefore, non- hemolytic bacteria are considered essential for exceptional probiotics. GJ231 was non- hemolytic on blood agar plates. In addition, the non- hemolytic enterotoxin (*Nhe*) and hemolysin BL (*Hbl*) genes were not identified in the GJ231 genome, which aligns with the strain’s lack of hemolytic activity. A potential risk for antibiotic resistance was the possibility of vertical or horizontal transfer. The GJ231 genome contains three antibiotic resistance genes: *InuA*, *ErmT*, and *ErmB*. Although there was a risk of gene transfer in GJ231, these genes are localized to genomic DNA rather than plasmid DNA, and therefore the risk was reduced. In addition, we annotated GJ231 with CRISPR-Cas, which prevents the horizontal transfer of virulence or antibiotic resistance genes, reducing the risk of gene transfer (Saidumohamed and Ganapathy Bhat, 2021). Analysis of potential metabolites in strain GJ231 did not reveal the presence of any toxic products.

After ingestion, bacteria are immediately exposed to the dual stresses of weak acids and bile salts. Therefore, acid and bile salt tolerance are essential criteria for selecting probiotics. Because the strong acid tolerance of LAB is not universal, strain-by-strain testing is required (Kailasapathy and Chin, 2000). Strain GJ231 exhibited both acid tolerance and bile salt tolerance. At weak acids, ATP synthase hydrolyses ATP to produce a transmembrane ionic gradient, which facilitates bacterial tolerance to strong acids (Deckers-Hebestreit and Altendorf, 1996). The GJ231 genome contains genes encoding ATP synthase and sodium antiporter proteins, as well as genes for L-lactate dehydrogenase, L-lactate permease, ATP-dependent *Clp*, and the phosphotransferase system. All of these components have mechanisms for providing resistance to weak acids and can work together to maintain pH homeostasis (Liang et al., 2023). Choloylglycine hydrolase (CBH), which hydrolyses the amide bond between glycine and bile acids, and inorganic pyrophosphatase, which affects the activity of bile acid coenzymes, were identified in the genome of *L. johnsonii* GJ231. Their presence implied a potential mechanism for the bile salt tolerance of strain GJ231 (Da et al., 1987; Lakshmi Ragavan and Das, 2021). The GJ231 genome also contained a variety of genes related to heat and stress tolerance, which echoed the results of the *in vitro* test. The heat-inducible transcriptional repressor *HrcA* and chaperone proteins (HslO, DnaJ, DnaJ, HtpX, HSP20, and HSP70) can help maintain the structure of proteins by preventing misfolding (Gladysheva et al., 2022; Chen et al., 2024). In addition, HSP had the ability to modulate the immune response (Huang et al., 2019). Under hyperosmotic conditions, the synergistic action of chaperone proteins (*dnaK* and *dnaJ*) encoded by the GJ231 genome contributes to repairing damage to the cellular macromolecular machinery (Schröder et al., 1993). *In vitro* assays revealed the excellent antioxidant capacity of strain GJ231. GJ231 also contained various oxidative stress-related enzymes scavenging of superoxide, peroxide, and hydroxyl radicals. These enzymes play a vital role in the antioxidant system and are closely related to the strain’s resistance to oxidative stress (Ezraty et al., 2017).

*In vivo experiments are the primary method used to evaluate the safety of probiotics* (Fan et al., 2023). A previous study reported that after administering *L. delbrueckii* subsp. *lactis* CIDCA to mice, no differences in body weight, feed intake, or organ indices were found in the group with CIDCA (de Jesus et al., 2022). The liver is an important organ responsible for metabolism and detoxification (Cui et al., 2023). In this study, the oral administration of GJ231 did not have a significant effect on body weight, food intake, or liver function (AST and ALT) in mice. This demonstrating that oral administration of GJ231 had no toxic effect on mice. However, we found the oral administration of GJ231 an increased thymic index in mice. The development of the thymus as a major lymphoid organ is vital for adaptive immunity (Kernfeld et al., 2018; Chen et al., 2019). SOD is the first line of defense against oxidative damage and plays a crucial role in oxidative stress and T-AOC is an oxidative stress marker (Zhao et al., 2021; Zhou et al., 2023). MDA is a byproduct of lipid peroxidation and has been utilized as a biomarker to indicate oxidative stress in organisms. In this study, the levels of serum T-AOC and SOD were significantly increased, while the levels of serum MDA was notably decreased in mice after administering GJ231. It suggested that the oral administration of GJ231 improved the mice’s ability to handle oxidative stress.

Probiotics have been reported to promote health and well-being in healthy dogs, e.g., as an adjunct product for the treatment of atopic dermatitis in dogs (Marsella, 2009; Kim et al., 2015) and to reduce the incidence of diarrhea (Nixon et al., 2019; Shmalberg et al., 2019). However, some studies have shown that supplemental feeding of *E. faecium* NCIB 10415 (isolated from the feces of healthy dogs) had no positive effect on the health status of dogs (Vahjen and Männer, 2003). Therefore, more studies are needed to discover the negative and positive effects of probiotics (canine or non-canine origin) on healthy and sick dogs to recycle probiotic products in dogs. Therefore, a strain with good probiotic potential (*Lactobacillus johnsonii* GJ231) was obtained in this study; larger studies are needed to explore the beneficial significance of the strain *Lactobacillus johnsonii* GJ231 at a broader level for prescription pet food and functional pet food development and/or improvement of canine intestinal health.

## 5 Conclusion

We isolated *L. johnsonii* GJ231 from the intestinal tract of healthy beagles. *In vitro* experiments revealed that GJ231 was tolerant of acid, bile salts, and heat. It was also capable of inhibiting the growth of various pathogens, had excellent free-radical scavenging activity, exhibited high levels of adherence, and possessed genes with the potential to biosynthesize a variety of bacteriocins. In conclusion, *L. johnsonii* GJ231 is a promising probiotic candidate for functional pet foods.

## Data Availability

The datasets presented in this study can be found in online repositories. The names of the repository/repositories and accession number(s) can be found in the article/Supplementary material.
