# Supplementary figures and images for: Genomic analysis and functional properties of Lactobacillus johnsonii GJ231 isolated from healthy beagles
Source: Front Microbiol. 2024 Sep 17;15:1437036. doi: 10.3389/fmicb.2024.1437036 (PMC11442259; doi:10.3389/fmicb.2024.1437036)

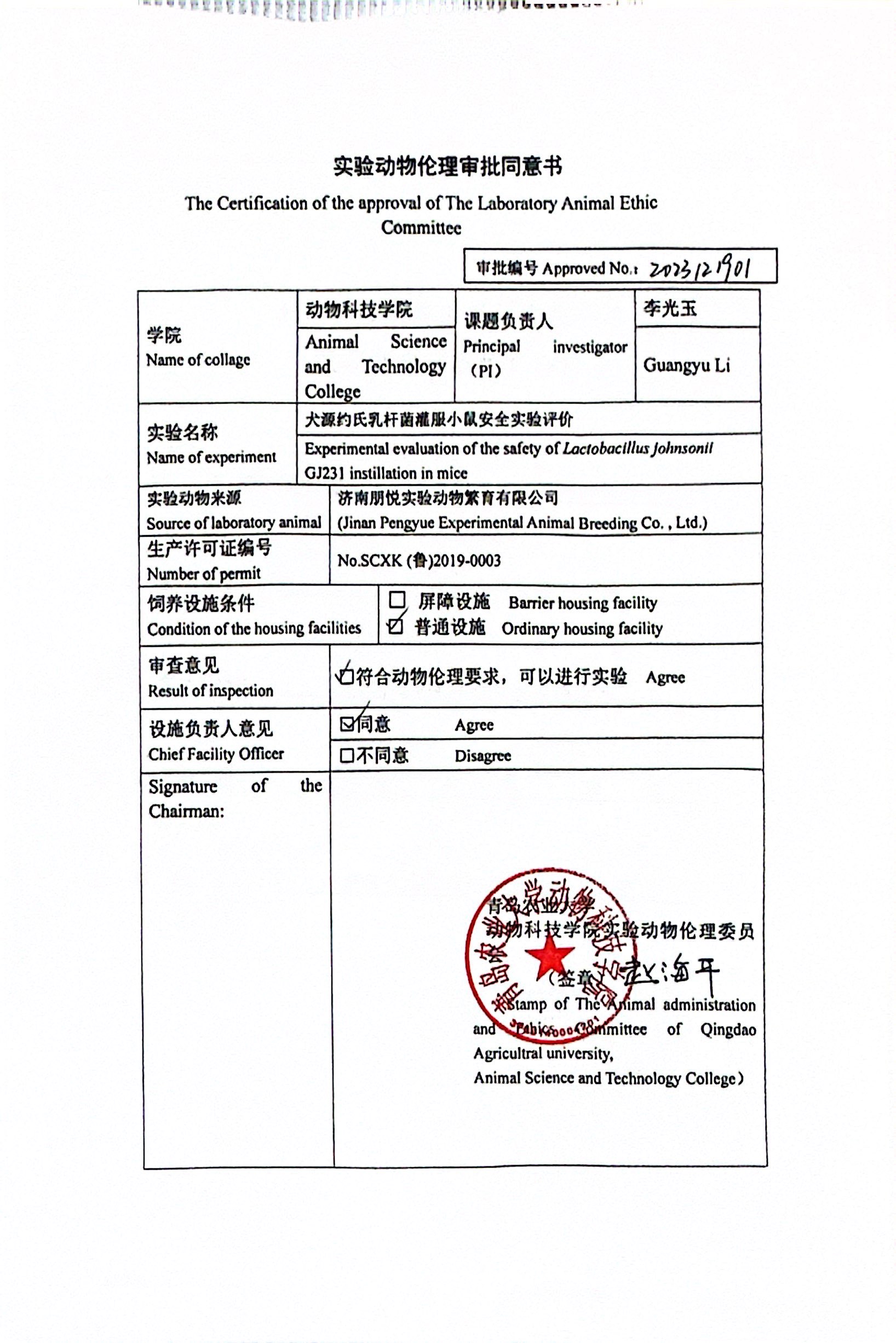

Supplement: Supplementary file 1 [file Image_1.JPEG]

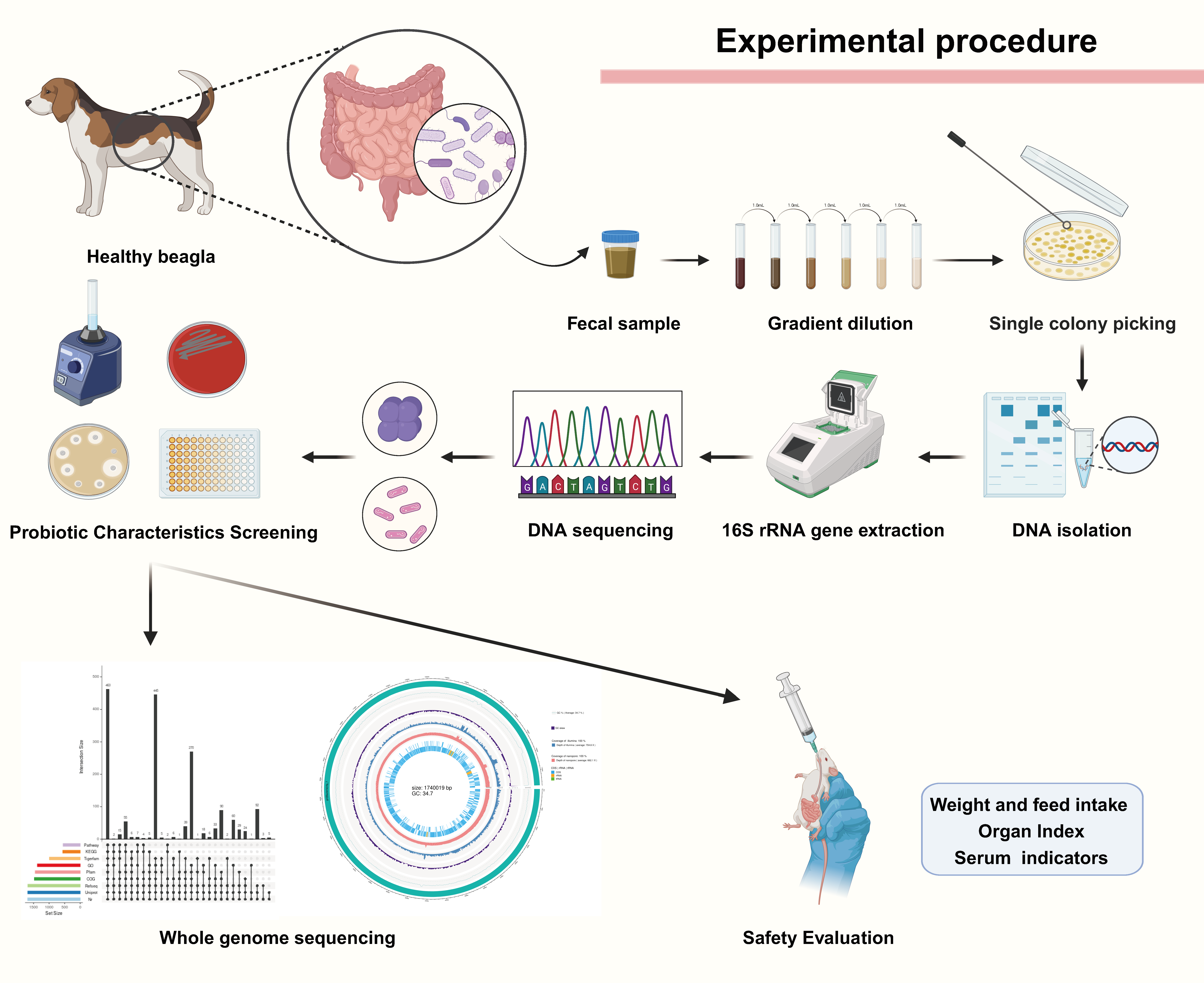

Supplement: Supplementary file 2 [file Image_2.TIFF]
