# Supplementary material for: Genomic analysis and functional properties of Lactobacillus johnsonii GJ231 isolated from healthy beagles
Source: Front Microbiol. 2024 Sep 17;15:1437036. doi: 10.3389/fmicb.2024.1437036 (PMC11442259; doi:10.3389/fmicb.2024.1437036)
Supplement: Supplementary file 3 [file Table_1.DOCX]

Supplementary Material

**STable 1.** Databases and web sites used for bioinformatic analyses.

| Database | Version | Website |
| --- | --- | --- |
| Uniport | UniProt 2022-03-09 | https://www.uniprot.org/ |
| PFAM | 35.0 | https://pfam.xfam.org/ |
| GO | 2022-03-09 | http://geneontology.org/ |
| KEGG | 2021-05-03 | https://www.kegg.jp/kegg/ |
| COG | 2022-03-09 | https://www.ncbi.nlm.nih.gov/COG/ |
| NR | 2022-03-09 | https://ftp.ncbi.nlm.nih.gov/blast/db/FASTA/ |
| ARDB | 1.1 | Ftp://ftp.cbcb.umd.edu/pub/data/ARDB/ |
| CAZy | 2021-09-24 | http://www.cazy.org/ |
| PHI | 4.12 | http://www.phi-base.org/ |
| VFDB | 2022-03-11 | http://www.mgc.ac.cn/VFs/ |
| TCDB | 2022-03-11 | http://www.tcdb.org/ |
| PLSDB | v.2021_06_23_v2 | https://ccb-microbe.cs.uni-saarland.de/plsdb/ |
| antiSMASH |  | https://antismash.secondarymetabolites.org/ |
| Bagel4 |  | http://bagel4.molgenrug.nl/ |
| SWISS-MODEL |  | https://swissmodel.expasy.org/ |

**STable 2.** Bacteriostatic result of *Lactobacillus johnsonii GJ231*.

| Items | Bacterial suspension GJ231 | Supernatant GJ231 |
| --- | --- | --- |
| *E. coli* | 13.57±1.59^a^ | 12.14±0.72^a^ |
| *S. aureus* | 18.71±1.08^b^ | 18.45±0.33^b^ |
| *Salmonella* | 14.75±0.51^a^ | 13.91±0.81^a^ |
| *L. monocytogenes* | 24.26±0.99^c^ | 23.14±0.76^c^ |
| *P. aeruginosa* | 19.58±0.84^b^ | 18.43±0.22^b^ |

Values are mean with SD.

**STable 3.** Genomics Islands prediction of *Lactobacillus johnsonii* GJ231.

| GI_ID | Seq_ID | Start | End | GI_Length |
| --- | --- | --- | --- | --- |
| GI1 | chromosome | 984350 | 989139 | 4790 |
| GI2 | chromosome | 1018920 | 1031885 | 12966 |
| GI3 | chromosome | 1047485 | 1054120 | 6636 |

**STable 4.** Prophage prediction of *Lactobacillus johnsonii* GJ231.

| **PP_ID** | **Contig_ID** | **PP_start** | **PP_end** | **attL_start** | **attL_end** | **attR_start** | **attR_end** | **attL_sequence** | **attR_sequence** |
| --- | --- | --- | --- | --- | --- | --- | --- | --- | --- |
| pp1 | chromosome | 183448 | 184380 | 186272 | 186286 | 186292 | 186306 | AAAGAAGGCATCCC | GGGATGCCTTCTTT |
| pp2 | chromosome | 260882 | 262129 | 262206 | 262219 | 262239 | 262252 | AAGCCTATTAAAT | ATTTAATAGGCTT |
| pp3 | chromosome | 868578 | 869384 | 868543 | 868557 | 868559 | 868573 | AAAAAAGTAGGATT | AATCCTACTTTTTT |
| pp4 | chromosome | 914400 | 915470 | 913393 | 913407 | 913424 | 913438 | AAAAAACGATTTGA | TCAAATCGTTTTTT |
| pp5 | chromosome | 992031 | 992954 | 994316 | 994330 | 994342 | 994356 | AATGATATCATTAA | TTAATGATATCATT |
| pp6 | chromosome | 1028230 | 1029120 | 1026536 | 1026548 | 1029596 | 1029608 | TTCAGCCATTTT | TTCAGCCATTTT |
| pp7 | chromosome | 1047485 | 1048282 | 1046504 | 1046517 | 1047232 | 1047245 | TTAGTTGGTTTAG | TTAGTTGGTTTAG |
| pp8 | chromosome | 7714 | 8565 | 7552 | 7565 | 7828 | 7841 | GGAGAATTTTTAA | GGAGAATTTTTAA |

PP_start: the starting position of the phage on the contig; PP_end: the ending position of the phage on the contig; attL_sequence: the sequence of the site to the left of the original phage; attR_sequence: the site to the right of the original phage.

**STable 5.** Statistics of repeated sequence prediction of *Lactobacillus johnsonii* GJ231.

| **Items** | **Number** | **Length(bp)** | **Coverage (%)** |
| --- | --- | --- | --- |
| SINE | 7 | 482 | 0.03% |
| LINE | 5 | 300 | 0.02% |
| DNA | 2 | 120 | 0.01% |
| Simple repeat | 84 | 3746 | 0.21% |
| Low complexity | 34 | 1540 | 0.09% |
| Other | 57 | 11464 | 0.65% |
| Total | 189 | 17652 | 1.00% |

SINE is short interspersed element, LINE is long interspersed element, DNA is transposon, and Total is total repetitive sequence.

**STable 6.** Insertion sequence analysis of *Lactobacillus johnsonii* GJ231.

| **Id** | **IS name** | **IS family** | **Pident(%)** | **Length(bp)** | **Mismatch** | **Qstart** | **Qend** | **Bitscore** |
| --- | --- | --- | --- | --- | --- | --- | --- | --- |
| chromosome | ISLjo5 | IS200/IS605 | 98.478 | 2102 | 31 | 490863 | 492964 | 3703 |
| chromosome | ISLjo5 | IS200/IS605 | 98.478 | 2102 | 31 | 490863 | 492964 | 3703 |
| chromosome | IS1223 | IS3 | 98.193 | 1494 | 27 | 782862 | 784355 | 2610 |
| chromosome | IS1223 | IS3 | 98.193 | 1494 | 27 | 714139 | 715632 | 2610 |
| chromosome | IS1223 | IS3 | 98.193 | 1494 | 27 | 1185285 | 1186778 | 2610 |
| chromosome | IS1223 | IS3 | 98.193 | 1494 | 27 | 472870 | 474363 | 2610 |
| chromosome | IS1223 | IS3 | 98.193 | 1494 | 27 | 1421226 | 1422719 | 2610 |
| chromosome | IS1223 | IS3 | 98.193 | 1494 | 27 | 265782 | 267275 | 2610 |
| chromosome | IS1223 | IS3 | 98.193 | 1494 | 27 | 1664157 | 1665650 | 2610 |
| chromosome | IS1223 | IS3 | 98.193 | 1494 | 27 | 1692464 | 1693957 | 2610 |
| chromosome | IS1223 | IS3 | 98.148 | 54 | 1 | 270695 | 270748 | 95.3 |
| chromosome | ISLjo1 | IS30 | 99.832 | 1187 | 2 | 581014 | 582200 | 2182 |
| chromosome | ISLga1 | IS30 | 92.054 | 1183 | 86 | 581018 | 582196 | 1657 |
| chromosome | ISLjo2 | ISL3 | 80.425 | 424 | 67 | 971567 | 971982 | 309 |
| chromosome | ISLjo2 | ISL3 | 90.361 | 166 | 16 | 971973 | 972138 | 219 |
| chromosome | ISLjo2 | ISL3 | 97.917 | 48 | 1 | 359994 | 360041 | 84.2 |
| chromosome | ISLhe61 | IS3 | 86.567 | 134 | 18 | 360044 | 360177 | 148 |

**STable 7.** Cytochromes P450 predictions of *Lactobacillus johnsonii GJ231*.

| ID | Pident | Qcovhsp | SequenceID | ProteinID | HomologousFamilies | Superfamilies | GI | TaxonID | Species |
| --- | --- | --- | --- | --- | --- | --- | --- | --- | --- |
| ctg_00356 | 30.4 | 77.7 | 86444 | 63948 | 719 | 94 | 108862162 | 39947 | OryzasativaJaponicaGroup |
| ctg_00709 | 29.4 | 30.8 | 99190 | 74873 | 381 | 51 | 325089259 | 544711 | AjellomycescapsulatusH88 |

**STable 8.** Virulence factors predictions of *Lactobacillus johnsonii GJ231*.

| **SeqID** | **Pident** | **Qcovs** | **Accession** | **Annotation** |
| --- | --- | --- | --- | --- |
| ctg_01102 | 72.89 | 99 | VFG016490(gb\|NP_975163) | (tuf) elongation factor Tu [EF-Tu (VF0877) - Adherence (VFC0001)] [Mycoplasma mycoides subsp. mycoides SC str. PG1] |
| ctg_01246 | 68.66 | 99 | VFG018662(gb\|WP_012130507) | (eno) phosphopyruvate hydratase [Streptococcal enolase (VF1060) - Exoenzyme (VFC0251)] [Streptococcus gordonii str. Challis substr. CH1] |
| ctg_01253 | 68.59 | 98 | VFG000077(gb\|NP_465991) | (clpP) ATP-dependent Clp protease proteolytic subunit [ClpP (VF0074) - Stress survival (VFC0282)] [Listeria monocytogenes EGD-e] |
| ctg_01339 | 68.40 | 95 | VFG006826(gb\|NP_464902) | (lisR) two-component response regulator [LisR/LisK (VF0792) - Regulation (VFC0301)] [Listeria monocytogenes EGD-e] |
| ctg_00645 | 68.32 | 99 | VFG005865(gb\|WP_002262524) | (galU) UTP--glucose-1-phosphate uridylyltransferase GalU [Capsule (VF0144) - Immune modulation (VFC0258)] [Streptococcus mutans UA159] |
| ctg_00441 | 67.74 | 97 | VFG012103(gb\|WP_003514589) | (groEL) chaperonin GroEL [GroEL (VF0594) - Adherence (VFC0001)] [Clostridium thermocellum ATCC 27405] |
| ctg_01074 | 64.85 | 98 | VFG002182(gb\|WP_002376666) | (cpsI) UDP-galactopyranose mutase [Capsule (VF0361) - Immune modulation (VFC0258)] [Enterococcus faecalis V583] |
| ctg_01069 | 62.33 | 99 | VFG047041(gb\|WP_014548762) | (wbtL) glucose-1-phosphate thymidylyltransferase [LPS (VF0542) - Immune modulation (VFC0258)] [Francisella cf. tularensis subsp. novicida 3523] |
| ctg_00812 | 61.94 | 93 | VFG037116(gb\|WP_013448170) | (msrA/B(pilB)) trifunctional thioredoxin/methionine sulfoxide reductase A/B protein [MsrAB (VF0456) - Stress survival (VFC0282)] [Neisseria lactamica 020-06] |
| ctg_00829 | 61.72 | 73 | VFG026980(gb\|YP_005923858) | (sigA/rpoV) RNA polymerase sigma factor [SigA (VF0257) - Regulation (VFC0301)] [Mycobacterium tuberculosis RGTB423] |
| ctg_00769 | 59.05 | 91 | VFG043573(gb\|NP_219906) | (dnaK) chaperone protein DnaK [Adherence; porin (VF0713) - Adherence (VFC0001)] [Chlamydia trachomatis D/UW-3/CX] |
| ctg_01303 | 58.90 | 99 | VFG000080(gb\|NP_464522) | (clpE) ATP-dependent protease [ClpE (VF0073) - Stress survival (VFC0282)] [Listeria monocytogenes EGD-e] |
| ctg_01249 | 58.28 | 100 | VFG019077(gb\|WP_000260685) | (plr/gapA) type I glyceraldehyde-3-phosphate dehydrogenase [Streptococcal plasmin receptor/GAPDH (VF1042) - Adherence (VFC0001)] [Streptococcus pneumoniae Hungary19A-6] |
| ctg_00752 | 58.19 | 97 | VFG045688(gb\|WP_002294134) | (cpsA/uppS) undecaprenyl diphosphate synthase [Capsule (VF0361) - Immune modulation (VFC0258)] [Enterococcus faecium Aus0004] |
| ctg_01081 | 57.92 | 92 | VFG016416(gb\|WP_000060578) | (BT9727_RS25850) sugar transferase [Polysaccharide capsule (VF0659) - Immune modulation (VFC0258)] [Bacillus thuringiensis serovar konkukian str. 97-27] |
| ctg_00610 | 57.14 | 12 | VFG040758(gb\|WP_014411717) | (pat2) esterase of alpha/beta hydrolase family [Phospholipase A2 (VF0494) - Exotoxin (VFC0235)] [Rickettsia prowazekii GvV257] |
| ctg_01101 | 57.08 | 94 | VFG005547(gb\|WP_000107749) | (tig/ropA) trigger factor [Trigger factor (VF1056) - Stress survival (VFC0282)] [Streptococcus agalactiae NEM316] |
| ctg_00640 | 54.17 | 95 | VFG001373(gb\|WP_000758382) | (cps4I) capsular polysaccharide biosynthesis protein Cps4I [Capsule (VF0144) - Immune modulation (VFC0258)] [Streptococcus pneumoniae TIGR4] |
| ctg_01203 | 53.19 | 9 | VFG001795(gb\|BAC98831) | (cba) c protein beta antigen [-C protein (VF0277) - Immune modulation (VFC0258)] [Streptococcus agalactiae FM027022] |
| ctg_01267 | 52.67 | 94 | VFG032466(gb\|WP_014093697) | (lgt) prolipoprotein diacylglyceryl transferase [Lipoprotein diacylglyceryl transferase lgt (VF0790) - Post-translational modification (VFC0315)] [Listeria ivanovii subsp. ivanovii PAM 55] |
| ctg_00310 | 52.53 | 98 | VFG000079(gb\|NP_463763) | (clpC) endopeptidase Clp ATP-binding chain C [ClpC (VF0072) - Stress survival (VFC0282)] [Listeria monocytogenes EGD-e] |
| ctg_00929 | 52.48 | 98 | VFG019080(gb\|WP_000731918) | (slrA) peptidylprolyl isomerase [Streptococcal lipoprotein rotamase A (VF1047) - Adherence (VFC0001)] [Streptococcus pneumoniae CGSP14] |
| ctg_01451 | 52.31 | 98 | VFG016307(gb\|WP_001084657) | (galE) UDP-glucose 4-epimerase GalE [Polysaccharide capsule (VF0659) - Immune modulation (VFC0258)] [Bacillus cereus ATCC 14579] |
| ctg_00788 | 52.15 | 100 | VFG032519(gb\|WP_012986167) | (bsh) bile salt hydrolase [BSH (VF0350) - Stress survival (VFC0282)] [Listeria seeligeri serovar 1/2b str. SLCC3954] |
| ctg_01458 | 52.09 | 99 | VFG031960(gb\|WP_014092990) | (lap) Listeria adhesion protein Lap [Lap (VF0444) - Adherence (VFC0001)] [Listeria ivanovii subsp. ivanovii PAM 55] |
| ctg_01070 | 51.51 | 100 | VFG007659(gb\|WP_011261022) | (rmlB) dTDP-glucose 4,6-dehydratase [Capsular polysaccharide (VF0624) - Immune modulation (VFC0258)] [Vibrio fischeri ES114] |
| ctg_01068 | 51.14 | 85 | VFG007663(gb\|WP_011149276) | (rmlC) dTDP-4-dehydrorhamnose 3,5-epimerase [Capsular polysaccharide (VF0624) - Immune modulation (VFC0258)] [Vibrio vulnificus YJ016] |
| ctg_00785 | 50.84 | 96 | VFG005582(gb\|WP_002897814) | (eno) phosphopyruvate hydratase [Streptococcal enolase (VF1060) - Exoenzyme (VFC0251)] [Streptococcus sanguinis SK36] |
| ctg_01203 | 50.00 | 9 | VFG001795(gb\|BAC98831) | (cba) c protein beta antigen [-C protein (VF0277) - Immune modulation (VFC0258)] [Streptococcus agalactiae FM027022] |
| ctg_01203 | 50.00 | 9 | VFG001795(gb\|BAC98831) | (cba) c protein beta antigen [-C protein (VF0277) - Immune modulation (VFC0258)] [Streptococcus agalactiae FM027022] |
| ctg_00226 | 50.00 | 99 | VFG030724(gb\|WP_015308640) | (sugC) sn-glycerol-3-phosphate ABC transporter ATP-binding protein UgpC [Trehalose-recycling ABC transporter (VF0842) - Nutritional/Metabolic factor (VFC0272)] [Mycobacterium smegmatis JS623] |
| ctg_01706 | 49.40 | 93 | VFG000080(gb\|NP_464522) | (clpE) ATP-dependent protease [ClpE (VF0073) - Stress survival (VFC0282)] [Listeria monocytogenes EGD-e] |
| ctg_00718 | 49.25 | 84 | VFG011430(gb\|WP_002963616) | (acpXL) acyl carrier protein [LPS (VF0367) - Immune modulation (VFC0258)] [Brucella melitensis bv. 1 str. 16M] |
| ctg_00503 | 49.23 | 100 | VFG002085(gb\|WP_000084793) | (vasH) type VI secretion system regulatory protein VasH [VAS T6SS (VF0335) - Effector delivery system (VFC0086)] [Vibrio cholerae O1 biovar El Tor str. N16961] |
| ctg_01276 | 49.18 | 94 | VFG018674(gb\|WP_008809635) | (SGO_RS08445) undecaprenyl/decaprenyl-phosphate alpha-N-acetylglucosaminyl 1-phosphate transferase [Capsule (VF0144) - Immune modulation (VFC0258)] [Streptococcus gordonii str. Challis substr. CH1] |
| ctg_00960 | 48.62 | 100 | VFG032519(gb\|WP_012986167) | (bsh) bile salt hydrolase [BSH (VF0350) - Stress survival (VFC0282)] [Listeria seeligeri serovar 1/2b str. SLCC3954] |
| ctg_01794 | 48.50 | 99 | VFG048851(gb\|WP_015958700) | (gndA) NADP-dependent phosphogluconate dehydrogenase [Capsule (VF0560) - Immune modulation (VFC0258)] [Klebsiella pneumoniae subsp. pneumoniae MGH 78578] |
| ctg_00858 | 48.47 | 99 | VFG047710(gb\|WP_014547360) | (carB) carbamoyl phosphate synthase large subunit [Pyrimidine biosynthesis (VF0558) - Nutritional/Metabolic factor (VFC0272)] [Francisella cf. tularensis subsp. novicida 3523] |
| ctg_01109 | 48.44 | 28 | VFG043478(gb\|WP_005752194) | (PM_RS08640) ComEA family DNA-binding protein [ComE1 (VF1230) - Adherence (VFC0001)] [Pasteurella multocida subsp. multocida str. Pm70] |
| ctg_00753 | 48.13 | 100 | VFG045683(gb\|WP_002294135) | (cpsB/cdsA) phosphatidate cytidylyltransferase [Capsule (VF0361) - Immune modulation (VFC0258)] [Enterococcus faecium Aus0085] |
| ctg_01590 | 47.79 | 28 | VFG043441(gb\|WP_002287386) | (EFMU0317_RS16950) C40 family peptidase [Fibronectin-binding protein (VF0747) - Adherence (VFC0001)] [Enterococcus faecium U0317] |
| ctg_01013 | 46.92 | 95 | VFG050094(gb\|WP_000136887) | (hlyIII) hemolysin III family protein [Hemolysin III (VF0655) - Exotoxin (VFC0235)] [Bacillus cereus G9241] |
| ctg_01233 | 46.89 | 66 | VFG030675(gb\|WP_003875535) | (sugC) sn-glycerol-3-phosphate ABC transporter ATP-binding protein UgpC [Trehalose-recycling ABC transporter (VF0842) - Nutritional/Metabolic factor (VFC0272)] [Mycobacterium avium subsp. paratuberculosis K-10] |
| ctg_00070 | 46.35 | 97 | VFG031736(gb\|WP_011778105) | (regX3) two-component sensory transduction protein RegX [RegX3 (VF0858) - Regulation (VFC0301)] [Mycobacterium vanbaalenii PYR-1] |
| ctg_01057 | 46.27 | 36 | VFG045610(gb\|WP_014387145) | (esp) Enterococcal surface protein; Esp [Esp (VF0353) - Adherence (VFC0001)] [Enterococcus faecium Aus0004] |
| ctg_01216 | 46.25 | 98 | VFG039536(gb\|NP_820549) | (CBU_1566) Coxiella Dot/Icm type IVB secretion system translocated effector [T4SS secreted effectors (VF0696) - Effector delivery system (VFC0086)] [Coxiella burnetii RSA 493] |
| ctg_00177 | 45.60 | 99 | VFG000670(gb\|NP_706258) | (gtrB) bactoprenol glucosyl transferase [LPS (VF0124) - Immune modulation (VFC0258)] [Shigella flexneri 2a str. 301] |
| ctg_00463 | 45.36 | 94 | VFG013265(gb\|WP_005694045) | (orfM) deoxyribonucleotide triphosphate pyrophosphatase [LOS (VF0044) - Immune modulation (VFC0258)] [Haemophilus influenzae Rd KW20] |
| ctg_01784 | 44.88 | 98 | VFG031404(gb\|WP_085981087) | (ctpV) copper-translocating P-type ATPase [Copper exporter (VF0849) - Nutritional/Metabolic factor (VFC0272)] [Mycobacterium intracellulare ATCC 13950] |
| ctg_00754 | 44.78 | 65 | VFG015002(gb\|WP_003138017) | (mucP) metalloprotease protease [Alginate (VF0091) - Biofilm (VFC0271)] [Pseudomonas aeruginosa UCBPP-PA14] |
| ctg_00951 | 44.56 | 96 | VFG018402(gb\|WP_000131305) | (mgtB) Mg2+ transport protein [MgtBC (VF0106) - Nutritional/Metabolic factor (VFC0272)] [Salmonella enterica subsp. arizonae serovar 62:z4,z23:-- str. RSK2980] |
| ctg_01715 | 44.53 | 99 | VFG032819(gb\|WP_014092401) | (dltA) D-alanine--poly(phosphoribitol) ligase subunit DltA [D-alanine-polyphosphoribitol ligase DltA (VF0795) - Post-translational modification (VFC0315)] [Listeria ivanovii subsp. ivanovii PAM 55] |
| ctg_00781 | 44.44 | 26 | VFG006042(gb\|WP_001222601) | (SAK_RS06335) LysR family transcriptional regulator [Capsule (VF0274) - Immune modulation (VFC0258)] [Streptococcus agalactiae A909] |
| ctg_01227 | 44.30 | 98 | VFG013515(gb\|WP_011961967) | (mrsA/glmM) phosphoglucosamine mutase [Exopolysaccharide (VF0755) - Immune modulation (VFC0258)] [Haemophilus influenzae PittEE] |
| ctg_01415 | 44.08 | 73 | VFG032046(gb\|WP_003743853) | (aut) autolysin [Auto (VF0348) - Invasion (VFC0083)] [Listeria monocytogenes SLCC2378] |
| ctg_00165 | 44.06 | 30 | VFG032054(gb\|WP_012985336) | (aut) autolysin [Auto (VF0348) - Invasion (VFC0083)] [Listeria seeligeri serovar 1/2b str. SLCC3954] |
| ctg_01083 | 43.93 | 94 | VFG016435(gb\|WP_001207181) | (BALH_RS26250) CpsD/CapB family tyrosine-protein kinase [Polysaccharide capsule (VF0659) - Immune modulation (VFC0258)] [Bacillus thuringiensis str. Al Hakam] |
| ctg_00708 | 43.88 | 94 | VFG006797(gb\|WP_011702610) | (stp) Stp1/IreP family PP2C-type Ser/Thr phosphatase [Serine-threonine phosphatase stp (VF0787) - Post-translational modification (VFC0315)] [Listeria welshimeri serovar 6b str. SLCC5334] |
| ctg_00075 | 43.87 | 98 | VFG005533(gb\|WP_002262650) | (htrA/degP) trypsin-like peptidase domain-containing protein [Serine protease (VF1055) - Exoenzyme (VFC0251)] [Streptococcus mutans UA159] |
| ctg_00918 | 43.62 | 94 | VFG006813(gb\|WP_011702633) | (lspA) signal peptidase II [Lsp (VF0351) - Post-translational modification (VFC0315)] [Listeria welshimeri serovar 6b str. SLCC5334] |
| ctg_01170 | 43.48 | 71 | VFG050255(gb\|NP_219986) | (CT_473) hypothetical protein [TTSS secreted effectors (VF0711) - Effector delivery system (VFC0086)] [Chlamydia trachomatis D/UW-3/CX] |
| ctg_00976 | 42.76 | 96 | VFG026398(gb\|WP_031599964) | (lysA) diaminopimelate decarboxylase [Lysine synthesis (VF0815) - Nutritional/Metabolic factor (VFC0272)] [Mycobacterium abscessus subsp. bolletii str. GO 06] |
| ctg_00814 | 42.57 | 80 | VFG037116(gb\|WP_013448170) | (msrA/B(pilB)) trifunctional thioredoxin/methionine sulfoxide reductase A/B protein [MsrAB (VF0456) - Stress survival (VFC0282)] [Neisseria lactamica 020-06] |
| ctg_01319 | 42.31 | 98 | VFG026433(gb\|YP_005360830) | (glnA1) glutamine synthetase [Glutamine synthesis (VF0816) - Nutritional/Metabolic factor (VFC0272)] [Mycobacterium tuberculosis RGTB327] |
| ctg_00261 | 42.19 | 80 | VFG005775(gb\|WP_000403526) | (cylA) ABC (ATP-binding cassette) transporter CylA [-haemolysin/cytolysin (VF0279) - Exotoxin (VFC0235)] [Streptococcus agalactiae NEM316] |
| ctg_01213 | 41.82 | 54 | VFG042844(gb\|WP_011010863) | (CPE_RS11870) type II secretion system GspH family protein [Type IV pili (VF0731) - Adherence (VFC0001)] [Clostridium perfringens str. 13] |
| ctg_00807 | 41.67 | 98 | VFG027322(gb\|WP_015306641) | (relA) Probable GTP pyrophosphokinase RelA (ATP:GTP 3'-pyrophosphotransferase) (PPGPP synthetase I) ((P)PPGPP synthetase) (GTP diphosphokinase) [RelA (VF0287) - Regulation (VFC0301)] [Mycobacterium smegmatis JS623] |
| ctg_01200 | 41.56 | 99 | VFG016424(gb\|WP_000276321) | (manA) mannose-6-phosphate isomerase, class I [Polysaccharide capsule (VF0659) - Immune modulation (VFC0258)] [Bacillus thuringiensis str. Al Hakam] |
| ctg_00700 | 41.54 | 96 | VFG016516(gb\|WP_011386956) | (hlyA) TlyA family RNA methyltransferase [Hemolysin (VF0879) - Exotoxin (VFC0235)] [Mycoplasma capricolum subsp. capricolum ATCC 27343] |
| ctg_00792 | 41.36 | 77 | VFG045346(gb\|WP_011149697) | (IlpA) immunogenic lipoprotein A [IlpA (VF0513) - Adherence (VFC0001)] [Vibrio vulnificus YJ016] |
| ctg_01057 | 41.18 | 36 | VFG045610(gb\|WP_014387145) | (esp) Enterococcal surface protein; Esp [Esp (VF0353) - Adherence (VFC0001)] [Enterococcus faecium Aus0004] |
| ctg_00821 | 40.94 | 100 | VFG045566(gb\|WP_010948065) | (LPG_RS11860) Dot/Icm type IV secretion system effector [Dot/Icm T4SS secreted effectors (VF0798) - Effector delivery system (VFC0086)] [Legionella pneumophila subsp. pneumophila str. Philadelphia 1] |
| ctg_01026 | 40.91 | 97 | VFG043551(gb\|WP_010908478) | (ML_RS08565) HU family DNA-binding protein [ML1683 (VF0867) - Adherence (VFC0001)] [Mycobacterium leprae TN] |
| ctg_00371 | 40.89 | 89 | VFG016389(gb\|WP_000727070) | (BCE_RS25805) LytR family transcriptional regulator [Polysaccharide capsule (VF0659) - Immune modulation (VFC0258)] [Bacillus cereus ATCC 10987] |
| ctg_01556 | 40.79 | 94 | VFG018243(gb\|WP_005462534) | (luxS) S-ribosylhomocysteinase [AI-2 (VF0406) - Biofilm (VFC0271)] [Vibrio parahaemolyticus RIMD 2210633] |
| ctg_01082 | 40.78 | 99 | VFG050207(gb\|WP_000566214) | (BCAH187_RS26560) hypothetical protein [Polysaccharide capsule (VF0659) - Immune modulation (VFC0258)] [Bacillus cereus AH187] |
| ctg_00219 | 40.74 | 99 | VFG047265(gb\|WP_014715028) | (lpxA/glmU) UDP-N-acetylglucosamine pyrophosphorylase/glucosamine-1-phosphate N-acetyltransferase [LPS (VF0542) - Immune modulation (VFC0258)] [Francisella noatunensis subsp. orientalis str. Toba 04] |
| ctg_01376 | 40.73 | 92 | VFG032908(gb\|WP_014093538) | (prsA2) post translocation chaperone PrsA2 [PrsA2 (VF0449) - Post-translational modification (VFC0315)] [Listeria ivanovii subsp. ivanovii PAM 55] |
| ctg_00181 | 40.46 | 81 | VFG016389(gb\|WP_000727070) | (BCE_RS25805) LytR family transcriptional regulator [Polysaccharide capsule (VF0659) - Immune modulation (VFC0258)] [Bacillus cereus ATCC 10987] |
| ctg_00922 | 40.46 | 100 | VFG005186(gb\|YP_001198858) | (fbp54) fibronectin-bing protein Fbp54 [FBPs (VF0243) - Adherence (VFC0001)] [Streptococcus suis 05ZYH33] |
| ctg_01426 | 40.43 | 14 | VFG019124(gb\|WP_000573793) | (SPCG_RS01845) glycosyltransferase family 2 protein [Capsule (VF0144) - Immune modulation (VFC0258)] [Streptococcus pneumoniae CGSP14] |
| ctg_00857 | 40.16 | 98 | VFG047724(gb\|WP_012429026) | (carA) carbamoyl phosphate synthase small subunit [Pyrimidine biosynthesis (VF0558) - Nutritional/Metabolic factor (VFC0272)] [Francisella tularensis subsp. mediasiatica FSC147] |
| ctg_00720 | 40.10 | 55 | VFG016532(gb\|NP_975942) | (oppF) oligopeptide ABC transporter permease [Capsule (VF0881) - Immune modulation (VFC0258)] [Mycoplasma mycoides subsp. mycoides SC str. PG1] |

**STable 9.** CRISPR-Cas systems prediction of *Lactobacillus johnsonii* GJ231.

| **SeqID** | **Start position** | **End position** | **Length (bp)** | **Repetitive unit sequence** | **Genome %** |
| --- | --- | --- | --- | --- | --- |
| Chromosome | 1,536,939 | 1,537,124 | 185 | TTGCTGCAGTATTAGTTTGTACAGGTG | 0.0105 |
